# Preparation of a miR-155-activating nucleic acid nanoflower to study the molecular mechanism of miR-155 in inflammation

**DOI:** 10.1186/s10020-022-00495-4

**Published:** 2022-06-17

**Authors:** Wenxin Wang, Jie Geng, Xiaohan Wu, Jianguang Zhang, Chenna Zheng, Huachun Rao, Tianyu Li, Yong Diao, Huiyong Yang

**Affiliations:** 1 Department of Medical, Huaqiao University, Quanzhou, 362021 China; 2 Quanzhou Medical College, Quanzhou, 362011 China; 3 Laboratory Medicine, Quanzhou Orthopedic-Traumatological Hospital of Fujian Traditional Chinese Medicine University, Quanzhou, China; 4 Xiamen Institute for Food and Drug Quality Control, Xiamen, China

**Keywords:** Nucleic acid nanoflowers, miR-155, RNA activation, Inflammation

## Abstract

At present, the molecular mechanisms underlying inflammation remain unclear. In recent years, research on inflammation has focused on stimulating cell inflammation by using exogenous pro-inflammatory substances such as lipopolysaccharide (LPS) or inflammatory factors. To investigate the molecular mechanism of inflammation from a new perspective, we designed a nucleic acid nanoflowers (NFs) complex to directly activate inflammatory genes to study the inflammatory response without the need for external microbial factors to trigger an inflammatory response. An RNAa-type target gene-activated NFs was designed. Human umbilical vein endothelial cells (HUVECs) were transfected with NFs carrying small activating RNA (saRNAs) to directly co-activate microRNA (miR)-155 and *SHIP1* genes. After RNA activation (RNAa)-type NFs were transferred into HUVECs, the expression of miR-155 and pro-inflammatory and cancer-related factors increased, anti-inflammatory factors were reduced, cell proliferation increased, and cell migration was promoted. IL-1β protein levels were decreased and SHIP1 expression was downregulated. When miR-155 and its target SHIP1 were both activated, the expression of both was unaltered, maintaining cell homeostasis. This points towards miR-155 overexpression can trigger inflammation, and that miR-155 and its target genes act as a molecular switch role in the development of inflammation.

**Supplementary Information:**

The online version contains supplementary material available at 10.1186/s10020-022-00495-4.

## Introduction

Inflammation, a defensive response of living tissues to damage, involves the vascular system. Vascular endothelial cells (VECs) form the inner single-cell layer that lines all blood vessels. Vascular endothelial growth factor (VEGF) participates in the regulation and generation of new blood vessels through its specific effects on vascular endothelial cells. Binding of VEGF to its receptor (VEGF-R) on endothelial cells results in a signal transduction cascade, the release of growth- and inflammatory factors, and, ultimately, endothelial cell proliferation and migration. These processes eventually produce a large number of new blood vessels that operate as a physical barrier between the blood and tissues (Philipp et al. 2020). They can rapidly secrete various active substances to regulate cardiovascular function, and at inflammatory sites, they can synthesize and secrete chemotactic factors such as IL-8 and MCP-1 to attract leukocytes (Ding and Sun 2020; Chang et al. 2015; Macejova et al. 2019). Vascular proliferation can promote a variety of inflammatory and malignant diseases. Changing from short- to long-term inflammatory responses can result in the breakdown of immune tolerance and lead to major physiological changes of tissues, organs, and normal cells, thus increasing the risk of non-communicable diseases in the young and elderly. Long-term inflammation is also a contributory factor in the increase in morbidity and mortality of many degenerative diseases. The precise molecular mechanisms underlying inflammation remain unclear (Vilmont et al. 2012). To date, studies investigating the mechanisms underlying inflammation have largely used inflammation-stimulating factors, such as LPS, to stimulate target cells and induce inflammatory responses(Pauley et al. 2008; Rajasekhar et al. 2017; Nazari-Jahantigh et al. 2012; Jablonski et al. 2016; Yan et al. 2016; Smet et al. 2020; Yu et al. 2019; Sadri Nahand et al. 2020).

MicroRNAs (miRNAs) are endogenous, small (20–25 nucleotides), non-coding RNA molecules. These miRNA genes lie in intron regions of the genome and exon regions of non-coding genes. MiRNAs are transcribed from their host genes. They are involved in post-transcriptional gene regulation by binding directly to specific mRNA targets(Liu et al. 2020; Wen et al. 2018; Wang et al. 2021). Each miRNA can regulate multiple genes, and multiple miRNAs can act synergistically to regulate the same gene (Song et al. 2017; Taheri et al. 2020; Guo et al. 2021). MiRNAs are involved in the regulation of multiple immune responses, including the proliferation and differentiation of neutrophils and T and B cells, and in disease onset and development (Wang et al. 2021; Goodwin et al. 2020; Rodriguez et al. 2007; Szczepankiewicz et al. 2020). When the body is in a state of chronic inflammation, miRNA expression becomes dysregulated (Stanczyk et al. 2008; Zhang et al. 2020). The dysregulation of miRNA expression contributes to disease development and malignant transformation (Ma et al. 2020; Zhang et al. 2019a; Jiang et al. 2020; Oliveira et al. 2020). This has caused miRNAs to become a potential candidate target for the treatment and prevention of a wide range of human diseases. miR-155 is a classic inflammatory miRNA (Faraoni et al. 2009). It is highly expressed in immune cells that infiltrate the diseased tissue (Lu et al. 2019). Overexpression of miR-155 can lead to the release of pro-inflammatory cytokines and the downregulation of anti-inflammatory factors, whereas negative feedback regulates signal transduction to promote the continuation of inflammation (Mann et al. 2017). Previous research has demonstrated that miR-155 is involved in the inflammatory response, but they have not paid attention to its source. If there is no external inflammatory substance stimulation, only increasing the expression of miR-155, can it cause inflammation?

RNA interference (RNAi) technology is widely used for the regulatory control of RNA expression. In 2006, Li et al. (2006) discovered that small, double-stranded RNAs complementary to a gene promoter can specifically activate the expression of downstream genes. They termed this phenomenon RNAa. RNAa is a mechanism of gene activation mediated by saRNAs (Wang et al. 2018). RNAa can be used to restore gene function by activating the expression of endogenous target genes and has considerable potential applications in gene regulation, and epigenetics, and as a novel gene therapeutic intervention. RNAa technology is straightforward and rapid to use; For example—a shorter experimental period and low experimental costs than traditional gene overexpression technology, which provides many advantages for its use in experimental exploration. Currently Dar et al. (2018) have built an saRNA database (http://bioinfo.imtech.res.in/manojk/sarna/) for gene therapy research.

While RNAa has multiple advantages, similar to RNAi, the efficiency of saRNA delivery into cells is low. Currently, nucleic acid substances are mostly delivered into cells using viral or non-viral vectors as mediators. Viral vectors, such as adenoviruses and adeno-associated viruses, carry a risk for infection or have limited capacity, which limits their application. Non-viral vectors, mainly liposomes, are generally safe and have unlimited capacity, but their transfection efficiency is unsatisfactory and when used at high concentrations, they affect cell growth. Thus, an efficient and low-impact cell delivery system is urgently needed.

Recently, NFs have been developed for high-efficiency, non-toxic, and high-load nucleic acid delivery. Nucleic acid, as a biopolymer that stores genetic information in all organisms, has good biocompatibility and low immunogenicity and thus is a very suitable nanomaterial for use in vivo (Zhang et al. 2019b; Mokhtarzadeh et al. 2019). NFs are prepared by rolling circle replication using a designed DNA template with embedded functional moieties and primers to generate concatemeric DNA (Zhu et al. 2013). NFs have an adjustable size and a large specific surface area. Shi et al. found that microRNA-responsive release of Cas9/sgRNA from DNA nanoflowers can be used for cytoplasmic protein delivery and enhanced genome editing, and the internal DNF core not only acts as a Cas9/sgRNA carrier with miR-21-responsive sequence, but also encodes for tumor Cell-targeted MUC1 aptamer and able to trigger intracellular lysosomal escape (Shi et al. 2020). In comparison to DNA origami, NFs sequence design is simple and NFs preparation is facile and highly efficient. The use of NFs in biology has been studied extensively (Mei et al. 2015; Zhang et al. 2015; Hu et al. 2014; Kim et al. 2017). RNA nanoparticles have become a research focus in the biomedical field. Lee et al. (Lee et al. 2017) prepared polymeric siRNA nanoparticles for tumor-targeted delivery based on rolling circle transcription. By designing DNA template sequences that can be amplified using the same amplification program, large quantities of saRNA sequences can be synthetized in vitro in a relatively short time. Hui et al. constructed RNAi nanoflowers by RCT technology, and by infiltrating the DNA aptamer AS1411 with strong affinity for nucleolin (NCL) into the structure, endowed the nanoflowers with the ability to target the membrane of tumor cells with high expression of NCL. The effect enhances the uptake of siRNA, thereby contributing to the targeted therapy of tumor cells (Cheng et al. 2018). However, there is no report on the use of RNA nanoflowers to study the role of microRNA in disease.

In this study, the saRNA sequence that has been reported (number: VEGF-706) to effectively activate the expression of related genes is used. We embedded the saRNA in the designed linear template of rolling circle replication (RCT), optimized the reaction system and identified the best reaction system for the RCT process; we used different methods to transfer NFs into cells to compare the most stable and effective method for cell entry. Finally, the expression of the target gene *VEGF* was detected, which proved the applicability of the linear template we designed. The template, optimized reaction system, and cell entry method were used in the design of NFs that activate miR-155. These can activate the expression of miR-155 without the need for external microbial factors to trigger an inflammatory response and facilitate the exploration of the role of miR-155 in inflammation. We designed different DNA templates and used a single amplification scheme to synthesize a large amount of NF-RNAa sequence complexes in vitro in a short time. The complexes were optimized to allow highly efficient nucleic acid delivery into target cells. Upon activation of miR-155 expression in the cells, we evaluated the expression of inflammatory genes and analyzed the effect of miR-155 activation on the initiation, transformation, and progression of inflammation.

## Materials and methods

### Cell culture

HUVECs (a friendly gift from Lin Junsheng, Huaqiao University School of Medicine) were cultured in high-glucose Dulbecco’s modified Eagle’s medium (Biological Industries) supplemented with 10% (v/v) heat-inactivated fetal bovine serum (Biological Industries) and 1% (v/v) antibiotic cocktail (100 U/ml penicillin, and 100 mg/ml streptomycin, Biological Industries) in an incubator at 37 °C with 5% CO_2_.

### saRNA design

**Fig. 1:**
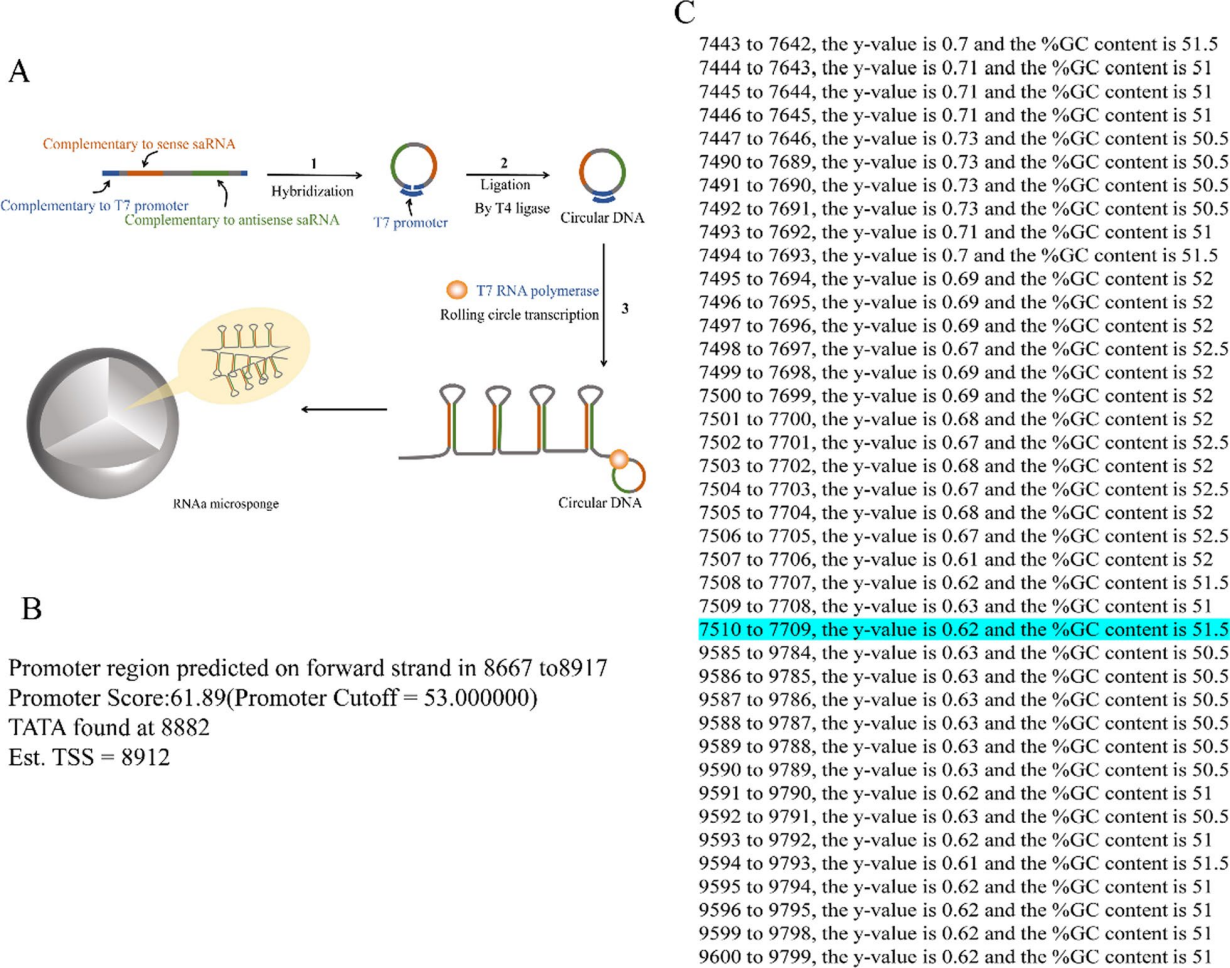
Design of NFs that can activate miR-155. **A** Schematic illustration of the preparation of NFs using rolling circle transcription (RCT). **B** The transcription start site of 10,000 bases upstream from the 5′end of the first exon of MiR155HG. **C** The enriched region of CpG islands

Based on information in the saRNA database and reports by Guo and Chen (Chen et al. 2011; Guo et al. 2016), we designed a linear DNA template (named L–T, with a phosphate group at the 5′ end) complementary to the T7 promoter primer (Additional file 4: Table S1) with embedded sense and antisense saRNA sequences (termed VEGF-706) that can effectively activate VEGF expression (Fig. 1A). Which proved that the usability of the linear template we designed and can be used to activate the miR-155 NFs.

The saRNAs were designed according to a report by Li et al. (2006). We searched for the sequence of the host gene, MiR155HG, and 10,000 bases upstream of the 5′ end of the first exon in databases (Additional file 1) such as NCBI (https://www.ncbi.nlm.nih.gov/gene/?term=) and UCSC (https://genome.ucsc.edu/), and we predicted the transcription start site to be located at base 8,912 (Fig. 1B) using various online resources (http://linux1.softberry.com/cgi-bin/programs/promoter/tssp.pl and http://www.bio-soft.net/sms/cpg_island.html). Next, we predicted the regions enriched in CpG islands to ensure that the designed saRNA avoids these regions (Fig. 1C). We found that the region of 7710–9584 was enriched in CpG islands (Fig. 1C). Therefore, the saRNA sense strand region of 7712–8712 was selected. We screened the sense strand region of the saRNA that can activate miR-155. Finally, seven eligible saRNAs were screened out (Table 1) and embedded into the template sequence designed above (Table 2).

**Table 1:** Sequences in line with saRNA design principles

Gene name	Positive-sense strand	Antisense strand
miR-155-1	GUC ACC UCA GCC UCC CAA A	CAG UGG AGU CGG AGG GUU U
miR-155-2	UAU CCC UCU UAG UCU GCU A	AUA GGG AGA AUC AGA CGA U
miR-155-3	GUC UGC UAG GGU UGC CAU A	CAG ACG AUC CCA ACG GUA U
miR-155-4	AUA GAC UGG AUG GCU GAU A	UAU CUG ACC UAC CGA CUA U
miR-155-5	ACA UUC UGG AGG CUA GAA A	UGU AAG ACC UCC GAU CUU U
miR-155-6	GGA CUC UCU UCC UGG CUU A	CCU GAG AGA AGG ACC GAA U
miR-155-7	UCU UCC UGG CUU ACA GGA A	AGA AGG ACC GAA UGU CCU U

**Table 2:** Template and primer sequence for preparing nucleic acid nanoflowers that can activate miR-155

Name	Sequence (5′ → 3′)
Template-1	ATAGTGAGTCGTATTAACGTACCAACAAGTCACCTCAGCCTCCCAAAACTTGTTTGGGAGGCTGAGGTGACATCCCT
Template-2	ATAGTGAGTCGTATTAACGTACCAACAATATCCCTCTTAGTCTGCTAACTTGTAGCAGACTAAGAGGGATAATCCCT
Template-3	ATAGTGAGTCGTATTAACGTACCAACAAGTCTGCTAGGGTTGCCATAACTTGTATGGCAACCCTAGCAGACATCCCT
Template-4	ATAGTGAGTCGTATTAACGTACCAACAAATAGACTGGATGGCTGATAACTTGTATCAGCCATCCAGTCTATATCCCT
Template-5	ATAGTGAGTCGTATTAACGTACCAACAAACATTCTGGAGGCTAGAAAACTTGTTTCTAGCCTCCAGAATGTATCCCT
Template-6	ATAGTGAGTCGTATTAACGTACCAACAAGGACTCTCTTCCTGGCTTAACTTGTAAGCCAGGAAGAGAGTCCATCCCT
Template-7	ATAGTGAGTCGTATTAACGTACCAACAATCTTCCTGGCTTACAGGAAACTTGTTCCTGTAAGCCAGGAAGAATCCCT
T7	TAATACGACTCACTATAGGGAT

All the above sequences (except T7) need to modify the phosphate group at the 5′end

The secondary structures of the products after 1 and 5 RCT cycles were analyzed using the M-fold website (http://unafold.rna.albany.edu/?q=mfold) (Additional file 3: Fig. S1). We found that it is better to produce multiple stem-loop structures. All templates were synthetized at GenScript (Nanjing, China). saRNAs were designed according to a report by Li et al. (2006). saRNAs that met all the inclusion criteria were selected and were embedded in the linear DNA template designed above to prepare nucleic acid NFs that can activate miR-155 (Additional files 1, 2).

### NFs production and characterization

The phosphorylated linear DNA template L–T and T7 Primer were mixed in 1 × T4 DNA ligase (2001A, TaKaRa) buffer (66 mM Tris–HCl, 6.6 mM MgCl_2_, 10 mM DTT, 66 μM ATP, and 3.3 μM [32P]-Na_4_P_2_O_7_). The DNA was denatured at 95 °C for 5 min and slowly cooled to room temperature to hybridize. Then, T4 DNA ligase was added at a final concentration of 10 U/µL to seal the nick of the linear template to form a circular template. The circular template (0.5 µM) was incubated with T7 RNA polymerase (5U/µL; M025, BioLabs) in 1 × T7 RNA polymerase buffer (40 mM Tris–HCl, 2 mM spermidine, 6 mM MgCl_2_, and 1 mM DTT) containing 2 mM rNTPs (4019, TaKaRa) and RNase Inhibitor (1 U/µL; R8060, Solarbio) at 37 °C for 24 h for RNA transcription and synthesis. After the reaction, the temperature was increased to 65 °C for 10 min to inactivate the polymerase. The reaction product was sonicated (40 kHz) for 3 min to prevent polymerization and then centrifuged at 12,000×*g* for 6 min. The supernatant was removed, and the pellet was resuspended in an equal volume of RNAse-free water to obtain purified NFs. To determine the NFs concentration, the absorbance at 260 nm was measured. NFs quality was assessed by 1% agarose gel electrophoresis. The samples were mixed with RNA loading buffer at a volume ratio of 4:1 and loaded on a gel. The electrophoresis conditions were 85 V, 200 A. The electrophoresis results were observed with a gel imaging system and images were captured.

Silicon (Si) wafers were soaked in aqua regia (3:1, H_2_SO_4_: HNO_3_) overnight, rinsed with ethanol and acetone, and dried. The purified nucleic acid NFs were dropped onto the Si wafers using a micropipette. The wafers were dried in an oven at 60 °C, gold-coated, and observed by scanning electron microscopy (SEM, Phenom) to examine NF morphology and size.

### Polyethylenimine (PEI)-NF complex preparation

One microgram of LPEI was diluted in RNase-free water to obtain a final volume of 10 μL and incubated at room temperature for 5 min. Further, 3 μg of centrifuged, purified NF from another centrifuge tube was diluted in RNase-free water to obtain a final volume of 10 μL and incubated at room temperature (25 ℃) for 5 min. The NFs (10 μL) were incubated with linear polyethylenimine (LPEI, Mw = 25,000) and diluted to 0.1 μg/μL in RNase-free water to form complexes (termed PEI-NFs) that can readily enter cells, then placed at room temperature for 15 min. Then, 3 μg of centrifuged purified NF was diluted with RNase-free water to a final volume of 20 μL and incubated at room temperature for 5 min. The same amount of the above two samples was taken (5 μL), and binding was assessed by gel electrophoresis.

### Cytotoxicity assay

Cells were seeded in 96-well plates at 5000 cells/well. The wells were assigned to a blank group, control group, and experimental group, with three replicate wells for each group, and the plates were incubated in a 5% CO_2_ incubator at 37 °C for 24 h. Then, the cells were washed with phosphate buffer saline (PBS) and the culture medium was replaced with 50 μL of fresh complete medium. PEI-NFs were added to the cultured cells and the plates were further incubated for 24 h. The cell counting kit-8 (C0038, Beyotime) assay was employed to detect cell viability. The experiment was repeated three times.

### Fluorescence cell imaging

We modified the T7 promoter sequence at the 5′end with the fluorophore Cy3 for fluorescent cell imaging to confirm NFs penetration into the cells. Cells were seeded into 24-well culture plates at 1 × 10^5^ cells/well and cultured in a 5% CO_2_ incubator at 37 °C for 24 h. PEI-NFs were added to the cells and the plates were further incubated for 24 h. Then, the cells were gently washed with PBS, fixed with 4% paraformaldehyde, permeabilized with 0.3% Triton X-100, and stained with DAPI (Beyotime). The stained cells were observed under an inverted fluorescence microscope (CKX41SF, Olympus Corporation).

### Flow-cytometric analysis of the efficiency of PEI-NF delivery into cells

HUVECs were seeded in 12-well plates at 1 × 10^5^ cells/well. The cells were cultured in a 5% CO_2_ incubator at 37 °C for 24 h. The cells were washed with PBS, the medium was replaced with fresh medium, PEI-NFs were added, and the plates were incubated for 12 h. Then, the cells were digested into a single-cell suspension. After centrifugation, the cells were washed several times with PBS and resuspended in 1 mL of PBS. Finally, the samples were subjected to flow cytometry.

### Cell scratch assay of cell migration

HUVECs were seeded into a 6-well plate at 2 × 10^5^ cells/well and incubated for 24 h. Then, the HUVECs were washed. Using a sterile 200-μL pipette tip, three scratches were made on each cell monolayer. The back of the 6-well plates was labeled with horizontal lines using marker pens for easy identification. The cells were washed twice, and complete medium was added. LPS and PEI-NFs were added to the cells. The scratches were evaluated at 12, 24, 48, and 72 h to compare the effects of the two treatments on cell migration capacity.

### Real-time reverse transcription RT-qPCR analysis of gene expression

Cells were seeded in a 24-well plate at 5 × 10^4^ cells/well. Wells were assigned to experimental groups and a control group. After 24 h of incubation, the PEI-NFs complexes were added. Total RNA was extracted at 12, 24, 48, and 72 h using an RNeasyTM Animal RNA Isolation Kit with spin columns (R0027, Beyotime) per the manufacturer’s protocol. The RNA was reverse-transcribed using HiScript^®^ Q RT SuperMix for qPCR (+ gDNA wiper) (Vazyme). The cDNA was used for PCR amplification of *VEGF, MiR55HG*, inflammation-associated genes, and DAPDH. In addition, the RNA was reverse-transcribed using the miRNA 1st Strand cDNA Synthesis Kit (by stem-loop) (MR101-01/02, Vazyme), a stem-loop primer for miR-155, and a reverse primer for U6 for reverse transcription of miR-155 and U6, respectively, and the cDNA was used for PCR amplification of miR-155 and U6. The primers used for RT-qPCR are listed in Table S2. The primers were synthetized at Nanjing Genscript (Nanjing, China). RT-qPCRs were run using the two-step method. Relative expression levels were determined using the 2^–ΔΔCt^ method.

### Western blotting

LPS/NFs-stimulated cells in 6-well plates were washed twice with ice-cold PBS. The cells were lysed with 200 μL of high-efficiency RIPA tissue/cell lysis buffer (R0010, Solarbio) on ice for 20–30 min. The lysates were centrifuged at 12,000×*g*, 4 °C for 5 min, and the supernatants were collected in centrifuge tubes and stored at − 80 °C. The total protein content was determined using an Enhanced BCA Protein Assay Kit (P0010S, Beyotime Biotechnology). Cell lysates containing 300 µg protein were mixed with 5 × sample buffer (containing DTT), and heated at 99 °C for 10 min. Then, the samples were separated by 12% SDS-PAGE at 70 V for 30 min followed by 120 V for 60 min and electroblotted on PVDF membranes at 300 mA for 1 h. The membranes were incubated in blocking solution (5% non-fat milk in TBST) at room temperature for 1 h. Then, the membranes were washed three times in TBST for 5 min each, incubated with primary antibodies (rabbit anti-SHIP1 polyclonal antibody, bs-3567R, Bioss and rabbit anti-GAPDH polyclonal antibody, bs-0755R, Bioss, diluted 1:1000 in TBST containing 5% BSA) at 4 °C overnight, washed with TBST buffer three times for 5 min each, and incubated with goat anti-rabbit IgG-HRP (1:2000, KGAA35, Keygen BioTECH) at room temperature for 1 h. The membranes were washed three times with TBST buffer for 10 min each. The membranes were exposed using BeyoECL Plus (P0018S, Beyotime), and band intensities were quantified using the ImageJ software.

### Prediction of miR-155 target genes and analysis of gene co-expression

We used four miR-155 target gene prediction tools, i.e., TargetScan (http://www.targetscan.org/vert_72/), miRanda (http://www.microrna.org/), PITA (https://pictar.mdc-berlin.de/), and picTar (https://pictar.mdc-berlin.de/), for miR-155 target gene prediction. For the predicted miR-155 target genes, we used the DAVID tool (https://david.ncifcrf.gov/tools.jsp) to analyze enriched signal pathways. The SHIP1 gene was selected, and miR-155 may promote PI3K-AKT signaling by inhibiting the expression of SHIP1, thereby causing inflammation. Thus, we designed nucleic acid NFs that can activate SHIP1 expression as described above to study its effect on miR-155 expression and explore the interaction between the miRNA and its target genes after activation.

### Data analysis and statistics

Data are expressed as mean ± standard deviation. For comparisons of multiple groups, one-way analysis of variance was used. Differences between two groups were compared using t-tests. Data were analyzed using the SPSS or Prism software (version 6.02, GraphPad Software). P < 0.05 was considered statistically significant.

## Results

### Production and characterization of the nucleic acid NFs

Based on information in the saRNA database and reports by Guo and Chen (2011; Guo et al. 2016), and according to a report by Li et al. (2006). Seven eligible saRNAs were screened out (Table 1) and embedded into the template sequence designed above (L–T) (Table 2) and NFs were successfully prepared (Fig. 1A).

**Fig. 2:**
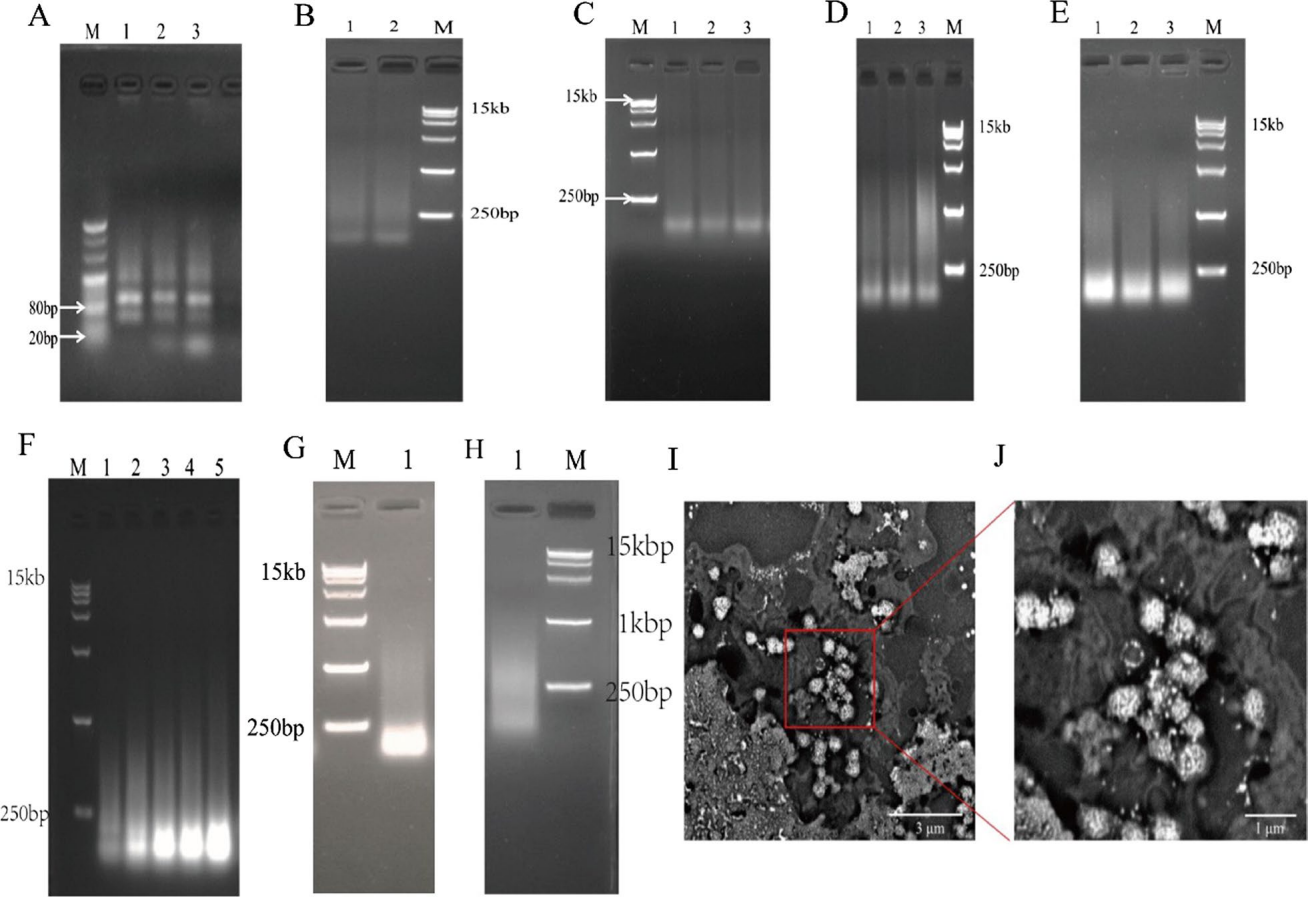
RCT reaction system optimization and NFs characterization. **A** Different concentration ratios of template and primer were used to perform the hybridization and ligation reactions. Lane M: DNA ladder, from top to bottom: 500 bp, 400 bp, 300 bp, 200 bp, 100 bp, 80 bp, and 20 bp; lanes 1–3: template to primer concentration ratios of 1:1, 1:2, and 1:3. **B** Various ligases were used for amplification. Lane M: DNA ladder, from top to bottom: 15 kb, 1 kb, 7500 bp, 5000 bp, 2500 bp, 1000 bp, and 250 bp; lane 1: Taq DNA ligase was used; lane 2: T4 DNA ligase was used. **C**–**E** Products amplified with different hybridization times and ligation times. Lane M: DNA ladder, from top to bottom, 15 kb, 1 kb, 7500 bp, 5000 bp, 2500 bp, 1000 bp, and 250 bp; **C** lanes 1–3: hybridization time 1 h, ligation time: 1 h, 2 h, 3 h. **D** Lanes 1–3: hybridization time 2 h, ligation time: 1 h, 2 h, 3 h. **E** Lanes 1–3: hybridization time 3 h, ligation time: 1 h, 2 h, 3 h. **F** Different concentrations of rNTPs were used for transcription. Lane M: DNA ladder, from top to bottom: 15 kb, 1 kb, 7,500 bp, 5,000 bp, 2,500 bp, 1,000 bp, and 250 bp; lanes 1–5: rNTP concentrations of 2 mM, 4 mM, 6 mM, 8 mM, and 10 mM. **G**, **H** Amplification products before and after rolling circle transcription system optimization. Lane 1: RNA; lane M: DNA ladder. **I**, **J** SEM images of the NFs

The experimental results after hybridization and ligation reaction with different ratios of template concentration and primer concentration are shown in Fig. 2A. According to lanes 1, 2, and 3, we found that product migration was blocked. With the increase of primer concentration, the remaining primers that are not bound to the template become more and more, and the band in the range of 20 bp becomes brighter and brighter. When the ratio of the template concentration to the primer concentration is 1:1, the primers are all bound to the template, and there is no remaining primer.

Select different DNA ligases for amplification, and the experimental results are shown in Fig. 2B. According to lanes 1 and 2, after using T4 DNA ligase and Taq DNA ligase for the ligation reaction, there is no difference in the size and brightness of the target band between the product and the subsequent RCT product, and the amplified products are all diffuse bands and the brightness ranging from 250 to 1000 bp were brighter.

Different hybridization times and different ligation times were used for amplification, and the experimental results are shown in Fig. 2C–E. According to lanes 1, 2, and 3, we found that when the hybridization time and ligation time were different, there was no difference in the size of the target band. However, the results of Fig. 2C show that the hybridization time is too long, which will affect the next step of the transcription process. Figure 2B shows that when the hybridization time is 2 h and the ligation time is changed, there is no difference in the size of the target band, but the brightness of lane 3 is brighter, and the concentration of non-specific products is also lower.

Different concentrations of rNTPs were used for transcription amplification, and the experimental results are shown in Fig. 2F. When other conditions are the same, with the increase of rNTP concentration, the brightness of the target band whose product size is above 250 bp does not change, but it gets brighter below 250 bp. It shows that when the concentration of raw materials increases, more and more raw materials are not consumed. We found that when the rNTP concentration was 2 mM and 4 mM, the brightness of the target band with a product size above 250 bp was similar, but the brightness became brighter below 250 bp.

Therefore, it was finally determined that the ratio of template concentration to primer concentration was 1:1 in subsequent experiments, and T4 DNA ligase was used for the ligation reaction. The hybridization time was 2 h, the ligation time was 3 h, and the rNTP concentration was 2 mM. The optimized result is shown in Fig. 2G, H, the size of the product is between 250 bp and 1 kb, and it is a diffuse band, which is consistent with the size reported in the literature. The size and morphology of NFs were characterized by SEM, and the experimental results are shown in Fig. 2I, J. As shown in Fig. 2I, we have successfully prepared nucleic acid nanoflowers, which are of homogenous sizes and shapes, and the size is nanoscale. From Fig. 2J, we can see that the self-folded surface of the NFs is petal-like, with a diameter of about 200 nm.

### PEI-NF complex and saRNA that can activate *VEGF* expression have no effect on cell activities

**Fig. 3.**
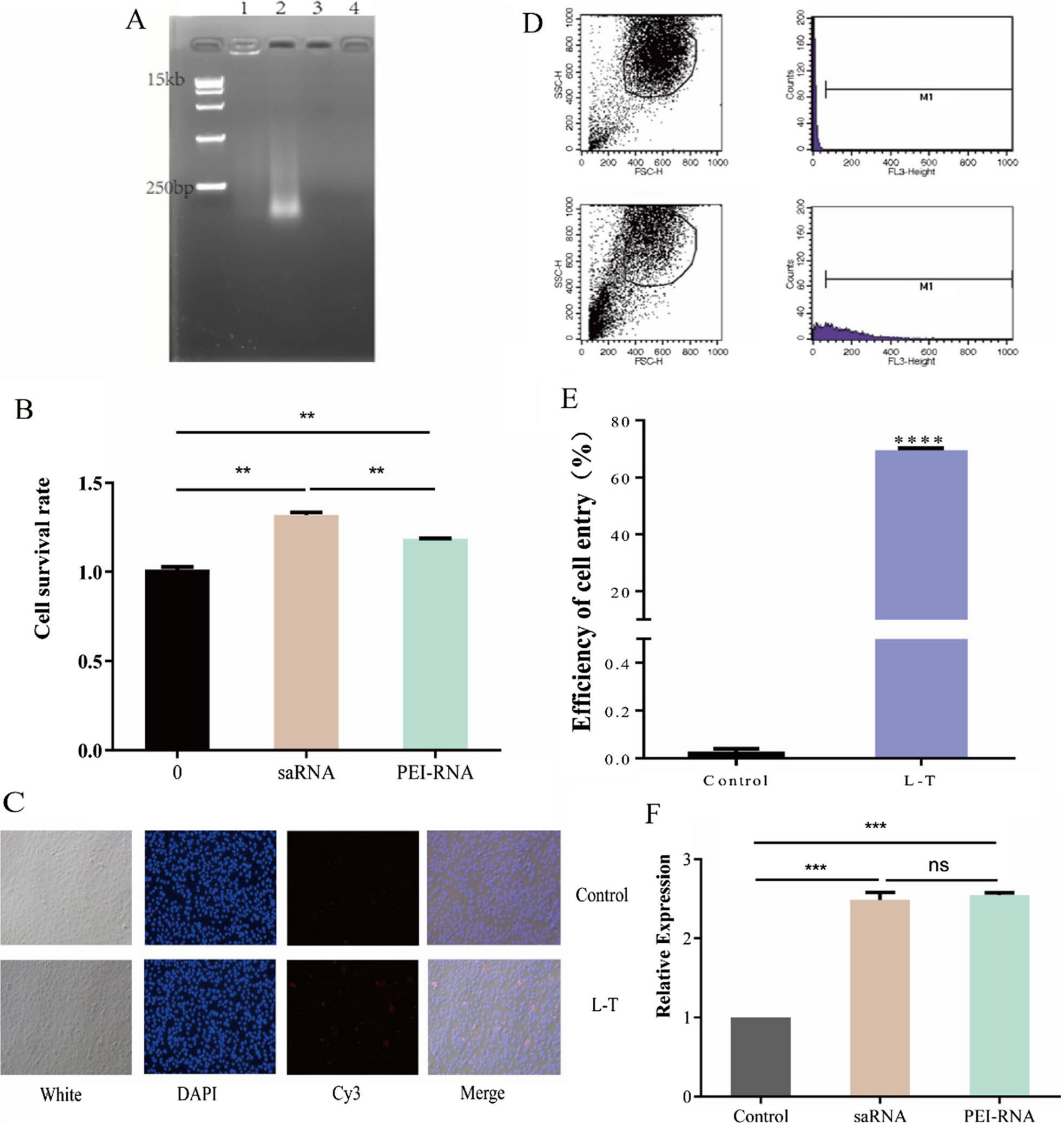
**A** Gel image of the product after the complexation of NFs and PEI. Lane 1: PEI-NFs complexes; lane 2: NFs; lane 3: PEI; lane 4: negative control (The loading amount was the same for both (5 µL), and the amount of nucleic acid was 0.15 µg/µL). **B** Cellular viability of HUVECs exposed to saRNA and PEI-NFs for 24 h. “0” represents the control. **C** Fluorescence imaging of cells treated with PEI-NF complex for 24 h (magnification, 10 ×). **D** Flow plots at 12 h after the NFs entered the cells. Results for the control group and experimental group are shown. **E** Efficiency of PEI-NF delivery into cells after 12 h (****P < 0.0001). **F** Relative VEGF expression in cells treated with saRNA or PEI-NFs complex for 72 h (**P < 0.01 vs control).

The results of gel imaging of the PEI-NF complexes are shown in Fig. 3A. According to lanes 1 and 2 in the figure, PEI and the nucleic acids interact via electrostatic interaction to form a positively charged complex, and the surface charge is reduced. Therefore, the target band does not migrate downward. When only PEI is present, bright bands do not appear in the absence of nucleic acids. Thus, the PEI-NF complex was successfully prepared.

The cytotoxicity of the PEI-NF complexes towards HUVECs was evaluated by CCK-8 assays (Fig. 3B). The survival rate of HUVECs treated with PEI-NFs or saRNA was > 1, these results showed that neither the PEI-NFs nor the saRNA had cytotoxic effects on HUVECs. Although the PEI-NFs group had a lower effect on cell proliferation than the saRNA group (P < 0.05), it had no inhibitory effect on cell proliferation compared with the control group, and PEI-NFs and saRNA had the same effect on gene activation in cells (Fig. 3F). This phenomenon may be because the saRNA is a double-stranded structure, which enters the cell and interacts with the AGO2 protein, releasing one of the two dsRNA strands, the passenger strand, and the remaining strand is called the guide strand, a complex consisting of guide-strand RNA, hnRNPs, and AGO2 is subsequently introduced into the nucleus, where it binds directly to DNA and promotes RNA-induced transcriptional activation. The PEI-RNA we designed is a long-chain RNA with a stem-loop structure. After entering the cell, the single-stranded and double-stranded parts are cleaved by corresponding enzymes. The cleaved single-strand is degraded, and the double-stranded RNA will exist stably. And promote RNA-induced transcriptional activation in cells in the same way as saRNA. Therefore, within 24 h, we see that the cell proliferation effect of PEI-RNA group will be lower than that of saRNA.

The cytotoxicity of the PEI-NF complexes towards HUVECs was evaluated by CCK-8 assays (Fig. 3B). The survival rate of HUVECs treated with PEI-NFs or saRNA was > 1, and there was no significant difference with the control group (P > 0.05). These results showed that neither the PEI-NFs nor the saRNA had cytotoxic effects on HUVECs. In addition, PEI-NFs and saRNA did not significantly suppress cell proliferation (P > 0.05). Thus, the PEI-NFs were further evaluated in subsequent experiments.

### PEI-NFs that can activate *VEGF* expression successfully enter cells

Fluorescence microscopy confirmed the presence of PEI-NFs within the cells (Fig. 3C). In the control group, no Cy3 fluorescence signal was detected, whereas in the experimental group (L–T), red fluorescence was observed, indicating that the PEI-NFs had successfully entered the cells. Flow cytometry was used to quantitatively evaluate the efficiency of PEI-NF delivery into the cells (Fig. 3D, E). Compared with the control group, the efficiency of PEI-NF delivery into the cells was > 70% (P < 0.0001; Fig. 3E).

### PEI-NF complex and saRNA activate *VEGF* expression

We examined the activation of *VEGF* expression induced by PEI-NF complex and saRNA using RT-qPCR (Fig. 3F). Compared with that in the control group, the relative expression of *VEGF* in the experimental group of cells treated with PEI-NFs or saRNA activated was increased by 2.5-fold (P < 0.01). This result showed that the PEI-NF complex and saRNA effectively activated *VEGF* expression. There was no difference in the relative *VEGF* expression between the PEI-NF- and saRNA-treated cells, indicating that they have similar efficiency. Thus, we confirmed that the NFs was effective and could be used for the activation of miR-155.

### Changes in the template sequences of miR-155-activating NFs have no impact on cell proliferation

**Fig. 4:**
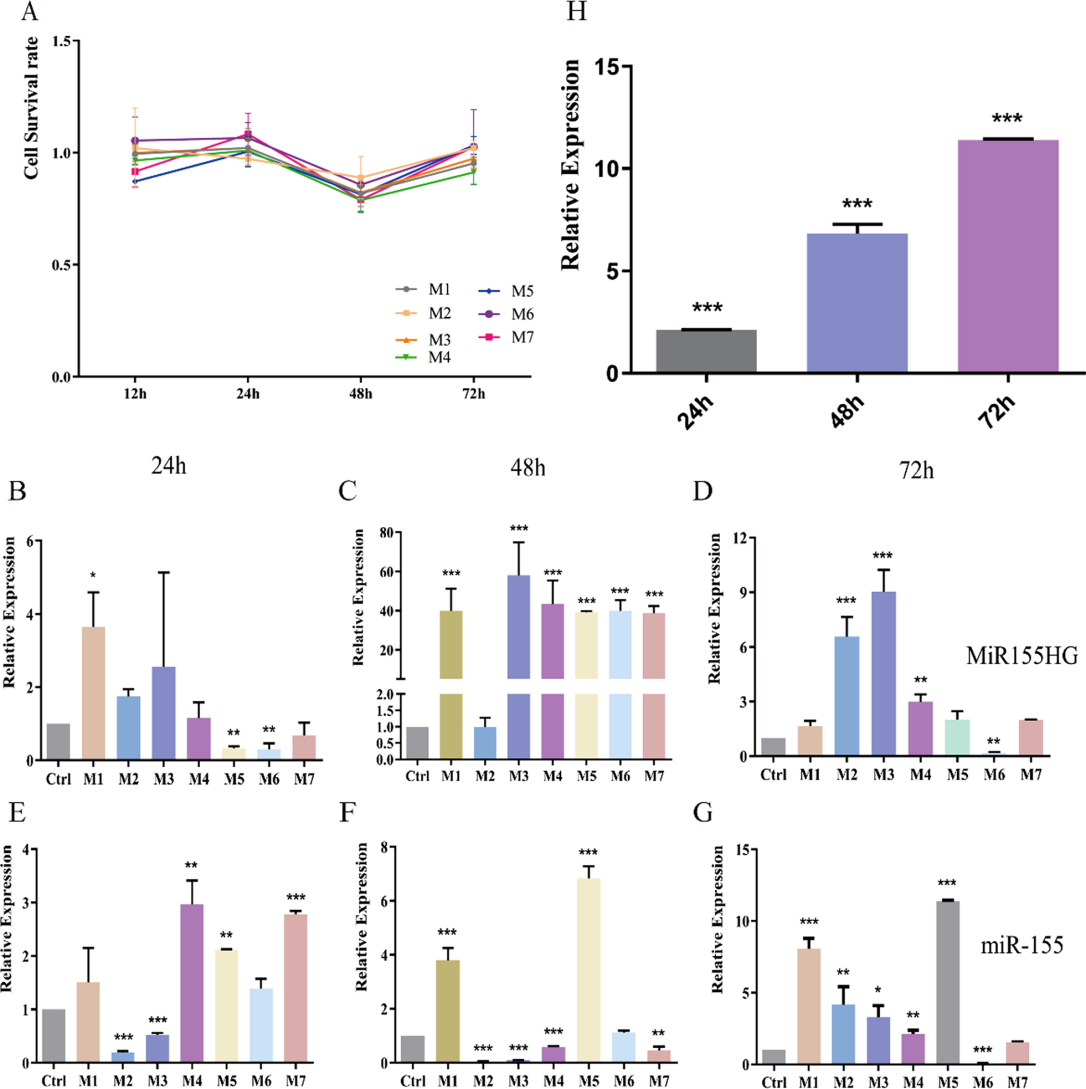
**A** NFs M1–M7 were prepared from different saRNA template sequences. Cell survival rates after 12, 24, 48, or 72 h were determined using the CCK-8 assay. P > 0.05 vs Ctrl. **B**–**D** Relative MiR155HG expression levels at 24 h, 48 h, or 72 h after treatment of HUVECs with NFs M1–M7. **E**–**G** Relative miR-155 expression levels at 24 h, 48 h, or 72 h after treatment of HUVECs with NFs M1–M7 (*P < 0.05 vs Ctrl; **P < 0.01 vs Ctrl; ***P < 0.001 vs Ctrl). (H) Relative miR-155 expression in HUVECs treated with M5 for 24 h, 48 h, or 72 h (*P < 0.05 vs Ctrl; **P < 0.01 vs Ctrl; ***P < 0.001 vs Ctrl)

In the above experiments, we screened seven saRNAs that can activate miR-155. We used the CCK-8 assay to detect the inhibitory effects of nucleic acid NFs M1–M7 prepared from seven different template sequences on cell proliferation at 12, 24, 48, and 72 h (Fig. 4A). Compared with the control group, NFs M1–M7 had no inhibitory effect on cell proliferation even after 72 h (P > 0.05). Nearly all NFs had similar effects, and the cell survival rate remained nearly constant from 12 to 24 h, slightly decreased between 24 and 48 h, and decreased from 48 to 72 h (to approximately 1). These findings showed that a change in the template sequence has no inhibitory effect on cell proliferation.

### Screening of saRNAs that can activate *miR-155* expression

To screen for saRNAs that can activate miR-155, we evaluated the NFs M1–M7 for their capacity to activate MiR155HG expression (Fig. 4B–D) and miR-155 expression (Fig. 4E–G) in HUVECs after treatment for 12, 24, 48, or 72 h. Compared with that in the control group (Ctrl), relative MiR155HG gene expression in the experimental groups treated with M1–M7 significantly increased within 48 h (P < 0.01) and decreased between 48 and 72 h. miR-155 expression increased over time. Based on the relative expression levels of miR-155, all seven saRNAs were found to activate miR-155 expression. M5 had the best effect; it enhanced miR-155expression level by 11.5-fold (P < 0.001) (Fig. 4H). Therefore, this saRNA was used to activate miR-155 expression in further experiments.

### NFs that can activate *miR-155* expression successfully enter cells

**Fig. 5:**
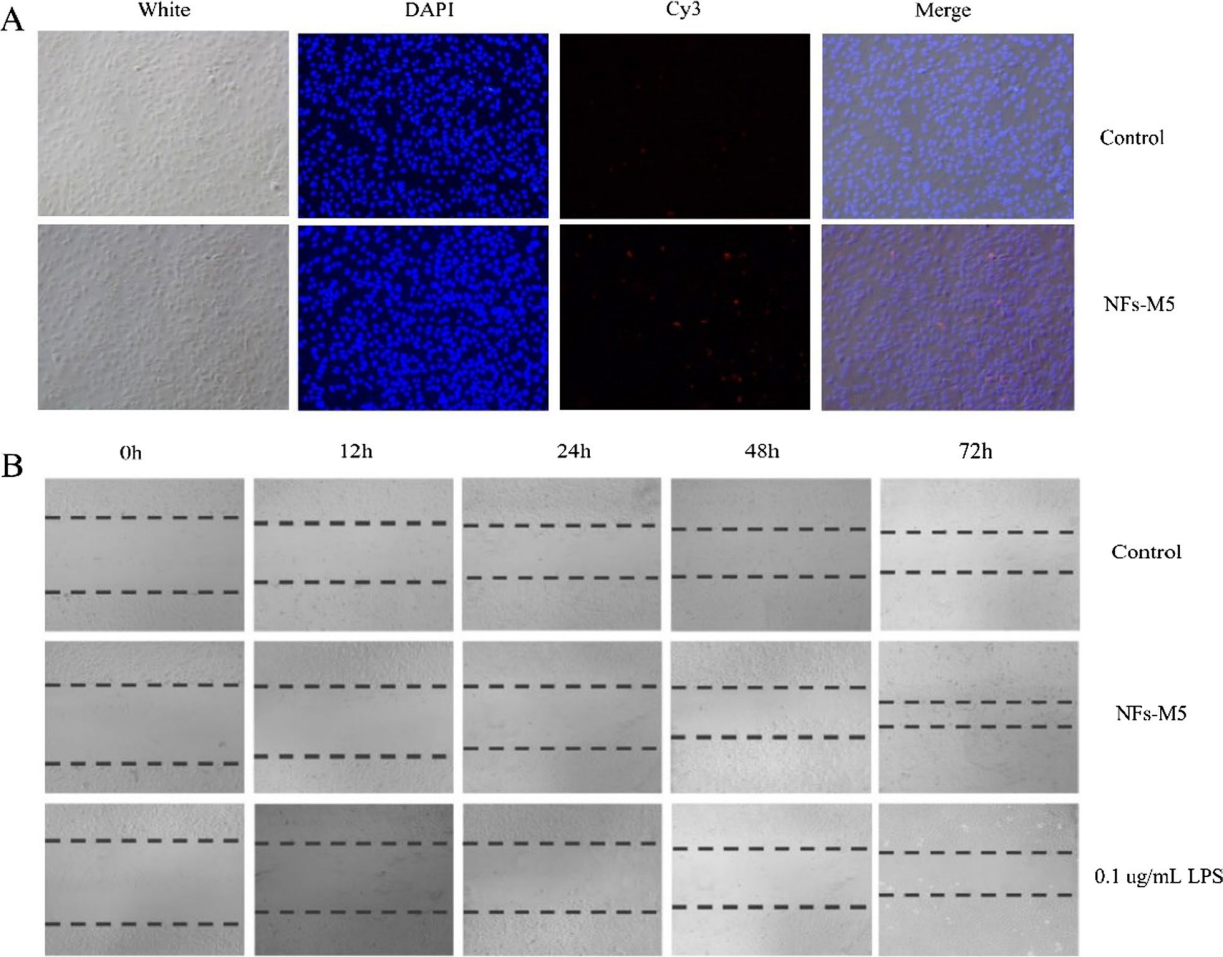
**A** Fluorescence imaging of cells after treatment with NFs (M5) for 24 h (magnification 10 ×). Cells stained with DAPI were imaged with an exposure time of 10 ms, and those stained with Cy3 were imaged with an exposure time of 100 ms. **B** Results of scratch assays of cells treated with NFs (M5) at 0 h, 12 h, 24 h, 48 h, and 72 h (10 ×)

Using fluorescence microscopy, we confirmed the presence of NFs that can activate miR-155 within cells (Fig. 5A). Through the images, we can find that the control group has no detected signal under the Cy3 microscope, while the experimental group (Template-5) can detect red fluorescence under the microscope, and after combining the pictures (Merge) we find that the red fluorescence is in the cells. Therefore, we can preliminarily conclude that the nucleic acid nanoflower M5 has successfully entered the cells.

### Overexpression of miR-155 promotes the migration of HUVECs

Cell migration was detected by a scratch assay at 0 h, 12 h, 24 h, 48 h, and 72 h after transfection of the cells (Fig. 5B). All cells migrated over time, but compared with the control group, cells with activated miR-155 expression migrated faster, indicating that miR-155 overexpression promoted cell migration as indicated by scratch closure. This result preliminarily indicated that miR-155 overexpression may cause inflammation.

### Activation of miR-155 affects the expression levels of key signaling molecules and inflammatory factors

**Fig. 6:**
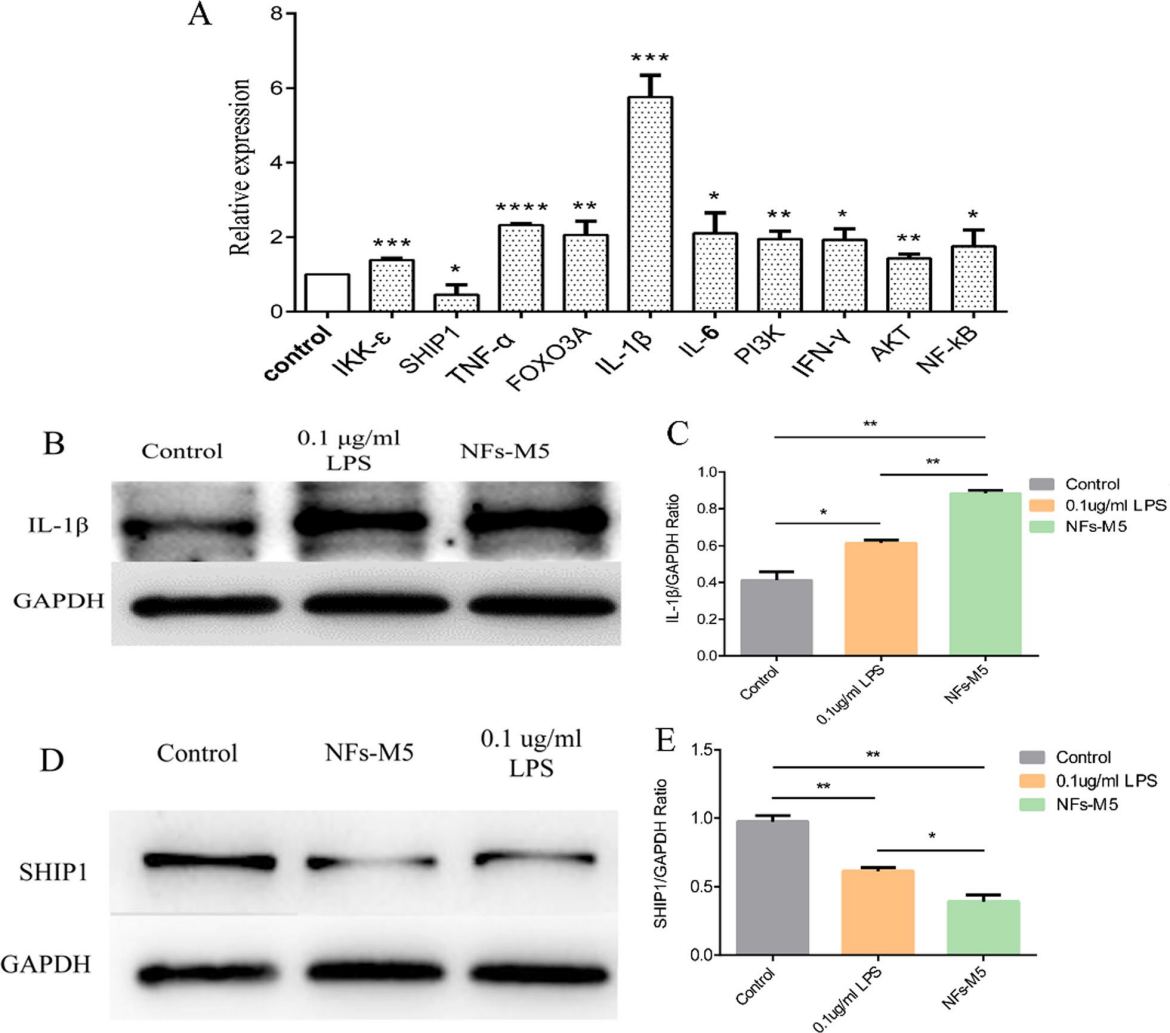
**A** Relative gene expression levels of inflammation-related factors (IKKɛ, SHIP1, TNF-α, FOXO3A, IL-1β, IL-6, PI3K, IFN-γ, AKT, and NF-κB) 72 h after treatment of HUVECs with NFs (M5) as determined by RT-qPCR. **B** Western blot analysis of IL-1β protein expression in cells treated with 0.1 μg/mL LPS or NF-M5 for 72 h. **C** Relative IL-1β protein levels (expression levels were normalized to that of GAPDH). **D** Western blot analysis of SHIP1 protein expression in cells treated with 0.1 μg/mL LPS or NF-M5 for 72 h. **E** Relative SHIP1 protein levels (expression levels were normalized to that of GAPDH)

In a preliminary experiment, we treated HUVECs with different concentrations of LPS and found that 0.1 μg/mL LPS effectively stimulated HUVECs; miR-155 expression first increased and then decreased, and the highest expression level was approximately fourfold higher than that in the control group (Additional file 3: Fig. S2A). Treatment of cells with a high concentration of LPS caused apoptosis (Additional file 3: Fig. S2B–H). Next, we studied whether activated miR-155 can trigger an inflammatory response. We treated HUVECs with the miR-155-activating NFs for 72 h and used RT-qPCR to detect changes in the expression of inflammation-related effectors and signaling pathway genes (Fig. 6A). After miR-155 activation, the relative expression levels of inflammation-related genes changed. Gene expression of the anti-inflammatory factor SHIP1 was significantly reduced (P < 0.01). Gene expression of the pro-inflammatory factors TNF-ɑ, IFN-γ, IL-1β, IL-6, and FOXO3A was significantly increased (P < 0.05). IKKɛ activity is related not only to inflammatory diseases, but also to cancer onset. IKKɛ may act as an oncogene promoting malignant transformation and tumor progression. Our research showed that after miR-155 was activated, IKKɛ gene expression was significantly increased (P < 0.001), suggesting that miR-155 activation may be related to cancer. The PI3K/AKT signaling pathway regulates multiple biological processes and is closely related with tumor development and metastasis (Cheng et al. 2020; Carnero et al. 2008). We found that PI3K/AKT gene expression increased after miR-155 activation (P < 0.01), which may be related to tumor development. NF-κB is a key transcription factor involved in inflammatory signaling pathways and responsible for the initiation of transcription of downstream inflammatory factors (Yu et al. 2020). Activated miR-155 increased the expression of NF-κB (P < 0.05) as well as that of pro-inflammatory factors (P < 0.05), indicating the induction of an inflammatory response. Together, these results suggested that miR-155 overexpression is related to inflammation and tumorigenesis.

### Activated miR-155 significantly upregulates IL-1β protein expression and downregulates SHIP1 protein expression

To verify that activation of miR-155 can induce inflammation, we treated HUVECs with miR-155-activating NFs for 72 h and then measured the expression of pro-inflammatory and anti-inflammatory proteins by western blotting. Compared with the control group, HUVECs treated with LPS or miR-155-activating NFs showed upregulated IL-1β protein expression and downregulated SHIP1 protein expression. However, compared with LPS, miR-155 activation had a significantly stronger promotive effect on IL-1β protein expression (P < 0.05, Fig. 6B, C) and suppressive effect on SHIP1 protein expression (P < 0.05, Fig. 6D, E). Thus, NF-M5 significantly induced the expression of the inflammatory factor IL-1β and reduced that of the negative regulator of inflammation, SHIP1. These results indicated that in the absence of exogenous inflammatory factors, NFs can directly activate miR-155 expression in cells and induce cell inflammation.

### Prediction of miR-155 target genes and analysis of gene co-expression

**Fig. 7:**
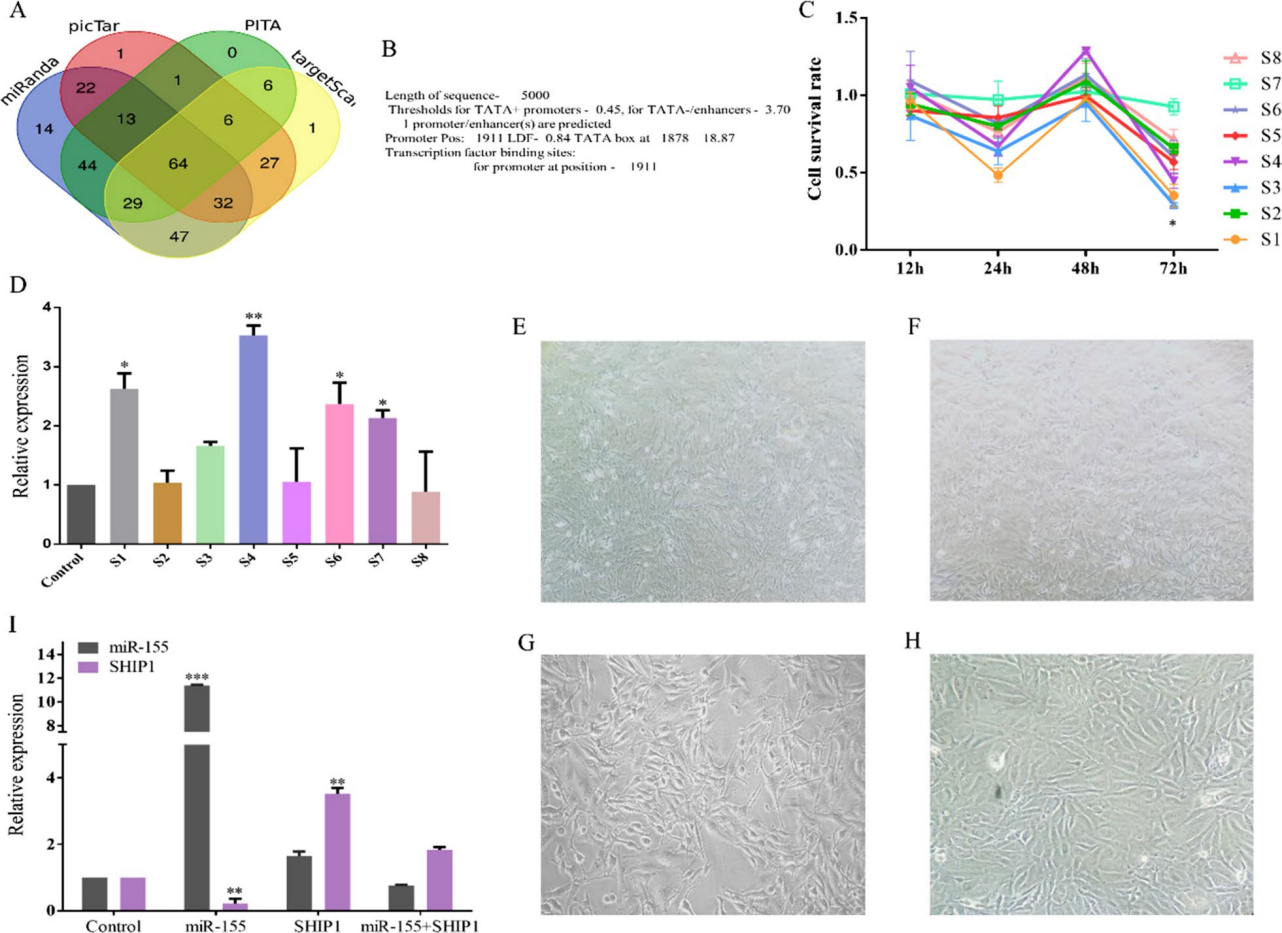
**A** Relationships between the numbers of miR-155 target genes predicted by the four tools used in this study. **B** Prediction of the transcription start site in the 5000 bases upstream of the 5' end of the first exon of INPP5D. **C** Cell survival rates of HUVECs treated with nucleic acid NFs S1–S8 activating SHIP1 at 12 h, 24 h, 48 h, and 72 h (*P < 0.05 vs. control). **D** SHIP1 expression in cells treated with nucleic acid NFs S1–S8 for 72 h (*P < 0.05 vs. control; **P < 0.01 vs. control). **E** Control cells cultured in the absence of NFs (magnification,4 ×). **F** Cell state at 72 h after miR-155 expression activation (magnification, 4 ×). **G** Cell state at 72 h after SHIP1 expression activation (magnification,10 ×). **H** Simultaneous activation of miR-155 and SHIP1 expression (magnification,10 ×). **I** Relative expression levels of SHIP1 and miR-155 in HUVECs treated with NF-M5, NF-S4, NF-M5, and NFs-S4 for 72 h (**P < 0.01 vs. control; ***P < 0.001 vs. control).

We predicted the human target genes of miR-155 using four miR-155 target gene prediction tools and analyzed the enriched regions of the target genes involved in the inflammatory signaling. The predicted target genes are listed in Additional file 4: Table S3. Although the numbers of target genes yielded by the different tools differed, there were a large number of common genes and only a few genes were predicted by only one tool. In total, 64 target genes were predicted by all four tools and thus had a high confidence (Fig. 7A). Therefore, these genes were selected as the final miR-155 target genes. Next, we used the DAVID tool to analyze the enrichment of these genes in signaling pathways, which revealed that the miR-155 target genes showed a distinct enrichment pattern. Forty-seven target genes were involved in 32 signaling pathways. There were eight groups of target genes involved in different inflammatory signaling pathways (Additional file 4: Table S4), suggesting that miR-155 regulates inflammatory signal transmission by targeting these genes to ultimately regulate the onset and development of inflammation. The PI3K-AKT pathway was enriched in miR-155 target genes. Our previous studies showed that the expression of the effectors SHIP1 and FOXO3A changed significantly after the activation of miR-155 expression (Additional file 3: Fig. S2B). This indicates that miR-155 may promote PI3K-AKT signaling by inhibiting the expression of SHIP1, thereby causing inflammation.

### Co-expression of miR-155 with its target SHIP1 cancels out their individual effects, suppressing inflammation

In the saRNA database, we did not find an saRNA that can activate SHIP1 expression. Therefore, according to the saRNA design principles reported by Li et al., we searched the 5000 bases upstream of the 5′ end of the SHIP1 gene, INPP5D (Additional file 2), for the transcription start site (Fig. 7B) and a region enriched in CpG islands. The transcription start site was at 1,911, and there was no region enriched in CpG islands. Therefore, the saRNA sense strand region (1–1711) was selected. We screened out eight saRNAs that met all the conditions (Additional file 4: Table S5), and embedded the selected sequences into the template sequence (Additional file 4: Table S6). We used the same methods as those used above to prepare NFs that can activate SHIP1 expression. The CCK-8 assay was used to evaluate the inhibitory effects of nucleic acid NFs S1–S8 prepared from the eight template sequences on cell proliferation at 12 h, 24 h, 48 h, and 72 h (Fig. 7C). Nucleic acid NFs S1–S8 had no inhibitory effect on cell proliferation in the first 48 h, but they did inhibit growth after 48 h (P < 0.05). All nucleic acid NFs had similar efficacy. This finding indicated that a change in SHIP1 expression affects cell proliferation and that SHIP1 expression is activated within 48 h to 72 h, inhibiting cell proliferation via the PI3K-AKT pathway.

Nucleic acid NFs S1–S8 activated SHIP1 expression after 72 h, as shown in Fig. 7D. S7 was the most effective. We evaluated cell morphology after the activation of miR-155 expression, SHIP1 expression, and both in HUVECs (Fig. 7E–H). In the presence of serum, the cell growth rate was high, and the cells were in good state and displayed long spindle-like morphology and tight connections (Fig. 7E). After miR-155 activation, the cell growth rate increased compared to that of control cells, the cells were in a normal state, cell density increased, and cells were slightly overlapping (Fig. 7F). Upon SHIP1 activation, the cell growth rate decreased, cell density was obviously reduced, and the cells showed a scattered distribution (Fig. 7G), suggesting that SHIP1 expression enhanced apoptosis in HUVECs. When miR-155 and SHIP1 expression was activated simultaneously, the cell growth rate was comparable to that of control cells and the cell density was only slightly decreased, indicating a normal state (Fig. 7H). Further, upon simultaneous activation of miR-155 and SHIP1, the expression of both genes did not change (Fig. 7I). These findings indicated that upon simultaneous activation of miR-155 and SHIP1, their individual effects are canceled out, and inflammation would not be promoted.

## Discussion

Changes in the inflammatory response from short-term to long-term, leading to a breakdown of immune tolerance and leading to major changes in all tissues and organs and normal cellular physiology, which increase the risk of various non-communicable diseases in young and old (Medzhitov 2021). In particular, systemic chronic inflammation (SCI) underlies cardiovascular disease, cancer, diabetes, chronic kidney disease, non-alcoholic fatty liver disease, autoimmune disease, and neurodegenerative diseases. MicroRNA is a kind of short single-stranded non-coding RNA, which is closely related to various human diseases. miR-155 is an important biomarker for understanding the molecular mechanism and etiology of various inflammatory diseases(Hu et al. 2022). Related studies have confirmed that miR-155 plays an important role in the regulation of uncontrolled inflammation and it may become a suitable target for disease treatment (Lu et al. 2019). Furthermore, the role that miR-155 plays in inflammatory response inhibition in the early stage of LPS-induced inflammation has been studied, and it is accepted that miR-155 exerts a pro-inflammatory effect in the middle and late stage (Liu et al. 2021). A miR-155 mimic or miR-155 containing exosomes inhibited cardiac fibroblast proliferation by downregulating Son of Sevenless 1 expression and promoted inflammation by decreasing Suppressor of Cytokine Signaling 1 expression (Wang et al. 2017). Studies have confirmed that persistent inflammation can lead to tumorigenesis, and the continuous increase of miR-155 can lead to persistent inflammatory response and promote tumorigenesis (Tili et al. 2011). Another in vitro study showed that serum exosome-derived miR-155 promoted macrophage proliferation and inflammation by targeting SHIP1 and SOCS1, respectively (Jiang et al. 2019). In vivo studies also suggest that inhibition of SHIP1 and SOCS1 by miR-155 modulates inflammatory responses (Mann et al. 2017). Thus, miR-155 is important for the occurrence and development of inflammatory diseases and cancer.

At present, most of the researches use pathogen-derived inflammatory factors or mimic to study inflammation. These are under the premise of triggering an inflammatory response to study the role of miR-155 in the inflammatory response. The premise of these studies is that the research subjects have already produced an inflammatory response, then miR-155 and other inflammatory factors will inevitably respond to this response. We can say that the inflammatory response triggers the changes in these factors. However, without the influence of exogenous factors, the activation of miR-155 can trigger an inflammatory response, which will provide us with a new perspective to understand the inflammatory response. Certain microRNAs are not only involved in the inflammatory response, but may also act as "switch molecules" for the inflammatory response. Their abnormal changes can provide early warning of major diseases, and provide new ideas for the prevention of inflammation, the treatment of related diseases, and the prevention of their deterioration.

Therefore, this study combined the RNAa method and NFs technology to precisely induce the expression of miR-155 from the promoter level in the absence of inflammatory responses caused by external microorganisms. The relationship between the increase of miR-155 expression and inflammation in normal environment was explored, and the effect of co-expression of miR-155 and its target genes on inflammation was analyzed. Thus, miRNA-155 was identified as an important "molecular switch" in the process of inflammation, in order to achieve early warning of major diseases, and provide new ideas for the prevention of inflammation, the treatment of related diseases and the prevention of its deterioration.

In this study, we successfully prepared NFs that can effectively activate miR-155, and verified that their efficacy was consistent with that reported in the literature (Fig. 3F). By evaluating the cell migration ability and the expression of inflammation-related factors after treatment with the NFs, we found that miR-155 expression was activated by NF-M5 stimulation, which promoted cell proliferation. Further, the activation of miR-155 expression promoted cell migration. The overexpression of miR-155 led to inflammation by activating the NF-κB signaling pathway, increasing the expression of pro-inflammatory factors, and inhibiting the expression of anti-inflammatory factors, and it also induced high expression of genes encoding tumor-related factors and genes involved in signaling pathways. Further study showed that miR-155 overexpression upregulated IL-1β and downregulated SHIP1 protein expression. Current research is consistent with this. IL-1β is an important mediator of the inflammatory process (Matsuoka et al. 2020). It promotes the release of neutrophils from the bone marrow, induces chemotaxis of monocytes and multinucleated cells to infiltrate the inflammation site, and induces local release lysosomal enzymes (Lübow et al. 2020; Mao et al. 2020; Sreejit et al. 2020). SHIP1 serves as an inhibitor of inflammation and NF-κB activity (Mann et al. 2017). It has been shown that miR-155, when upregulated in synovial tissue and synovial fluid macrophages, targets SHIP1, leading to increased levels of pro-inflammatory cytokines (Lu et al. 2019). Thus, our findings demonstrated that in the absence of exogenous inflammatory factors, direct activation of miR-155 expression can trigger cell inflammation and possibly, the occurrence and development of tumors. miR-155 can be regarded a switch molecule that triggers inflammation.

We explored the effect of co-expression of miR-155 and its target genes on inflammation. To this end, we first predicted and screened the target genes of miR-155. Upon co-expression of miR-155 and SHIP1, we found that miR-155 targeted SHIP1 and inhibited its expression, and SHIP1 promoted this effect of miR-155, so that ultimately, they canceled out each other’s expression and effects, which is expected to suppress inflammation.

## Conclusion

We combined our RNAa approach with NFs technology to develop an efficient method to directly activate inflammatory genes using nucleic acid nanoflower (NFs) complexes without the need for external microbial factors to trigger inflammatory responses. We demonstrate that under normal circumstances, miR-155 causes inflammation by increasing the expression of pro-inflammatory factors and suppressing the expression of anti-inflammatory factors. miR-155 can interact with its target genes after co-expression and affect the onset and development of inflammation. This study provided a new research idea for the molecular switch of inflammation. In disease research, miR-155 can be used as a marker to assess whether the body has inflammation and can provide important information for early disease detection and warning. This study also provides a promising strategy for targeting microRNAs and their target genes to treat diseases.

## Supplementary Information

**Additional file 1.** Host gene MiR155HG and the 10,000 base sequence upstream of the 5'end of the first exon.

**Additional file 2.** Gene INPP5D and the 5000 base sequence upstream of the 5'end.

**Additional file 3: Fig. S1**. The secondary structure of the product after 1 time (A) and 5 times (B) of rolling circle transcription. **Fig. S2**. (A) The expression of miR-155 after 48h of LPS treatment of cells with different concentrations. (B)Relative expression of inflammation-related factor genes 72h after NFs (M5) treatment of cells (C) The speciation diagram of HUVECs cell line under normal culture. (D-I) The speciation diagram of the HUVEC cell line stimulated by LPS at 0.1, 1, and 10μg/mL for 12 h. (F-H) The speciation diagram of the HUVEC cell line stimulated by LPS at 0.1, 1, and 10μg/mL for 24 h. (*P<0.05vs Control; **P<0.01 vs Control).

**Additional file 4: Table S1**. Template and primer sequence information for preparing NFs. **Table S2**. RT-qPCR amplification primer information. Table S3 miR-155 target gene prediction results. **Table S4.** Enrichment analysis of target genes of miR-155. **Table S5**. Sequences in line with saRNA design principles. **Table S6.** Template and primer sequence for preparing nucleic acid nanoflowers that can activate SHIP1.

## Data Availability

The datasets used and/or analysed during the current study are available from the corresponding author on reasonable request.

## Abbreviations

LPS: Lipopolysaccharide
NFs: A nucleic acid nanoflowers
HUVECs: Human umbilical vein endothelial cells
saRNAs: Small activating RNA
RNAa: RNA activation
VECs: Vascular endothelial cells
VEGF: Vascular endothelial growth factor
miRNAs: MicroRNAs
RNAi: RNA interference
RCT: Rolling circle replication
T7: T7 promoter primer
LPEI: Polyethylenimine linear
PBS: Phosphate buffer saline
CY3: Cyanine3 alkyne

## References

[CR1] Carnero A, Blanco-Aparicio C, Renner O, Link W, Leal JF (2008). The PTEN/PI3K/AKT signalling pathway in cancer, therapeutic implications. Curr Cancer Drug Targets.

[CR2] Chang H, Cao Y, Lin YI, Zhu H, Fu Y, Chen X, Zhang Q (2015). Association between toll-like receptor 6 expression and auxiliary T cells in the peripheral blood of pediatric patients with allergic purpura. Exp Ther Med.

[CR3] Chen R, Wang T, Rao K, Yang J, Zhang S, Wang S, Liu J, Ye Z (2011). Up-regulation of VEGF by small activator RNA in human corpus cavernosum smooth muscle cells. J Sex Med.

[CR4] Cheng H, Hong S, Wang Z, Sun N, Wang T, Zhang Y, Chen H, Pei R (2018). Self-assembled RNAi nanoflowers via rolling circle transcription for aptamer-targeted siRNA delivery. J Mater Chem B.

[CR5] Cheng J, Huang Y, Zhang X, Yu Y, Wu S, Jiao J, Tran L, Zhang W, Liu R, Zhang L (2020). TRIM21 and PHLDA3 negatively regulate the crosstalk between the PI3K/AKT pathway and PPP metabolism. Nat Commun.

[CR6] Dar SA, Kumar M (2018). saRNAdb: resource of small activating RNAs for up-regulating the gene expression. J Mol Biol.

[CR7] De Smet EG, Van Eeckhoutte HP, Avila Cobos F, Blomme E, Verhamme FM, Provoost S, Verleden SE, Venken K, Maes T, Joos GF (2020). The role of miR-155 in cigarette smoke-induced pulmonary inflammation and COPD. Mucosal Immunol.

[CR8] Ding T, Sun J (2020). Mechanistic understanding of cell recognition and immune reaction via CR1/CR3 by HAP-and SiO(2)-NPs. Biomed Res Int.

[CR9] Faraoni I, Antonetti FR, Cardone J, Bonmassar E (2009). miR-155 gene: a typical multifunctional microRNA. Biochim Biophys Acta.

[CR10] Goodwin AJ, Li P, Halushka PV, Cook JA, Sumal AS, Fan H (2020). Circulating miRNA 887 is differentially expressed in ARDS and modulates endothelial function. Am J Physiol Lung Cell Mol Physiol.

[CR11] Guo X, Feng L, Jia J, Chen R, Yu J (2016). Upregulation of VEGF by small activating RNA and its implications in preeclampsia. Placenta.

[CR12] Guo H, Pu M, Tai Y, Chen Y, Lu H, Qiao J, Wang G, Chen J, Qi X, Huang R (2021). Nuclear miR-30b-5p suppresses TFEB-mediated lysosomal biogenesis and autophagy. Cell Death Differ.

[CR13] Hu R, Zhang X, Zhao Z, Zhu G, Chen T, Fu T, Tan W (2014). DNA nanoflowers for multiplexed cellular imaging and traceable targeted drug delivery. Angew Chem Int Ed Engl.

[CR14] Hu J, Huang S, Liu X, Zhang Y, Wei S, Hu X (2022). miR-155: an important role in inflammation response. J Immunol Res.

[CR15] Jablonski KA, Gaudet AD, Amici SA, Popovich PG, Guerau-de-Arellano M (2016). Control of the inflammatory macrophage transcriptional signature by miR-155. PLoS ONE.

[CR16] Jiang K, Yang J, Guo S, Zhao G, Wu H, Deng G (2019). Peripheral circulating exosome-mediated delivery of miR-155 as a novel mechanism for acute lung inflammation. Mol Ther.

[CR17] Jiang L, Qiao Y, Wang Z, Ma X, Wang H, Li J (2020). Inhibition of microRNA-103 attenuates inflammation and endoplasmic reticulum stress in atherosclerosis through disrupting the PTEN-mediated MAPK signaling. J Cell Physiol.

[CR18] Kim E, Zwi-Dantsis L, Reznikov N, Hansel CS, Agarwal S, Stevens MM (2017). One-pot synthesis of multiple protein-encapsulated DNA flowers and their application in intracellular protein delivery. Adv Mater.

[CR19] Lee JH, Ku SH, Kim MJ, Lee SJ, Kim HC, Kim K, Kim SH, Kwon IC (2017). Rolling circle transcription-based polymeric siRNA nanoparticles for tumor-targeted delivery. J Control Release.

[CR20] Li LC, Okino ST, Zhao H, Pookot D, Place RF, Urakami S, Enokida H, Dahiya R (2006). Small dsRNAs induce transcriptional activation in human cells. Proc Natl Acad Sci USA.

[CR21] Liu C, Li N, Liu G (2020). The role of MicroRNAs in regulatory T cells. J Immunol Res.

[CR22] Liu Y, Wan X, Yuan Y, Huang J, Jiang Y, Zhao K, Wang Y, Liu Y, Wang Q, Jin H (2021). Opposite effects of miR-155 in the initial and later stages of lipopolysaccharide (LPS)-induced inflammatory response. J Zhejiang Univ Sci B.

[CR23] Lu Q, Wu R, Zhao M, Garcia-Gomez A, Ballestar E (2019). miRNAs as therapeutic targets in inflammatory disease. Trends Pharmacol Sci.

[CR24] Lübow C, Bockstiegel J, Weindl G (2020). Lysosomotropic drugs enhance pro-inflammatory responses to IL-1β in macrophages by inhibiting internalization of the IL-1 receptor. Biochem Pharmacol.

[CR25] Ma L, Zhang Y, Hu F (2020). miR-28-5p inhibits the migration of breast cancer by regulating WSB2. Int J Mol Med.

[CR26] Macejova D, Podoba J, Toporova L, Grigerova M, Kajo K, Machalekova K, Brtko J (2019). Causal associations of autoimmune thyroiditis and papillary thyroid carcinoma: mRNA expression of selected nuclear receptors and other molecular targets. Oncol Lett.

[CR27] Mann M, Mehta A, Zhao JL, Lee K, Marinov GK, Garcia-Flores Y, Lu LF, Rudensky AY, Baltimore D (2017). An NF-κB-microRNA regulatory network tunes macrophage inflammatory responses. Nat Commun.

[CR28] Mao L, Kitani A, Hiejima E, Montgomery-Recht K, Zhou W, Fuss I, Wiestner A, Strober W (2020). Bruton tyrosine kinase deficiency augments NLRP3 inflammasome activation and causes IL-1β-mediated colitis. J Clin Invest.

[CR29] Matsuoka T, Yoshimatsu G, Sakata N, Kawakami R, Tanaka T, Yamada T, Yoshida Y, Hasegawa S, Kodama S (2020). Inhibition of NLRP3 inflammasome by MCC950 improves the metabolic outcome of islet transplantation by suppressing IL-1β and islet cellular death. Sci Rep.

[CR30] Medzhitov R (2021). The spectrum of inflammatory responses. Science.

[CR31] Mei L, Zhu G, Qiu L, Wu C, Chen H, Liang H, Cansiz S, Lv Y, Zhang X, Tan W (2015). Self-assembled multifunctional DNA nanoflowers for the circumvention of multidrug resistance in targeted anticancer drug delivery. Nano Res.

[CR32] Mokhtarzadeh A, Vahidnezhad H, Youssefian L, Mosafer J, Baradaran B, Uitto J (2019). Applications of spherical nucleic acid nanoparticles as delivery systems. Trends Mol Med.

[CR33] Nazari-Jahantigh M, Wei Y, Noels H, Akhtar S, Zhou Z, Koenen RR, Heyll K, Gremse F, Kiessling F, Grommes J (2012). MicroRNA-155 promotes atherosclerosis by repressing Bcl6 in macrophages. J Clin Invest.

[CR34] Oliveira SR, Dionísio PA, Correia Guedes L, Gonçalves N, Coelho M, Rosa MM, Amaral JD, Ferreira JJ, Rodrigues CMP (2020). Circulating inflammatory miRNAs associated with Parkinson's disease pathophysiology. Biomolecules.

[CR35] Pauley KM, Satoh M, Chan AL, Bubb MR, Reeves WH, Chan EK (2008). Upregulated miR-146a expression in peripheral blood mononuclear cells from rheumatoid arthritis patients. Arthritis Res Ther.

[CR36] Philipp J, Le Gleut R, Toerne CV, Subedi P, Azimzadeh O, Atkinson MJ, Tapio S (2020). Radiation response of human cardiac endothelial cells reveals a central role of the cGAS-STING pathway in the development of inflammation. Proteomes.

[CR37] Rajasekhar M, Olsson AM, Steel KJ, Georgouli M, Ranasinghe U, Brender Read C, Frederiksen KS, Taams LS (2017). MicroRNA-155 contributes to enhanced resistance to apoptosis in monocytes from patients with rheumatoid arthritis. J Autoimmun.

[CR38] Rodriguez A, Vigorito E, Clare S, Warren MV, Couttet P, Soond DR, van Dongen S, Grocock RJ, Das PP, Miska EA (2007). Requirement of bic/microRNA-155 for normal immune function. Science.

[CR39] Sadri Nahand J, Moghoofei M, Salmaninejad A, Bahmanpour Z, Karimzadeh M, Nasiri M, Mirzaei HR, Pourhanifeh MH, Bokharaei-Salim F, Mirzaei H (2020). Pathogenic role of exosomes and microRNAs in HPV-mediated inflammation and cervical cancer: a review. Int J Cancer.

[CR40] Shi J, Yang X, Li Y, Wang D, Liu W, Zhang Z, Liu J, Zhang K (2020). MicroRNA-responsive release of Cas9/sgRNA from DNA nanoflower for cytosolic protein delivery and enhanced genome editing. Biomaterials.

[CR41] Song Z, Zhang L, Wang Y, Li H, Li S, Zhao H, Zhang H (2017). Constitutive expression of miR408 improves biomass and seed yield in Arabidopsis. Front Plant Sci.

[CR42] Sreejit G, Abdel-Latif A, Athmanathan B, Annabathula R, Dhyani A, Noothi SK, Quaife-Ryan GA, Al-Sharea A, Pernes G, Dragoljevic D (2020). Neutrophil-derived S100A8/A9 amplify granulopoiesis after myocardial infarction. Circulation.

[CR43] Stanczyk J, Pedrioli DM, Brentano F, Sanchez-Pernaute O, Kolling C, Gay RE, Detmar M, Gay S, Kyburz D (2008). Altered expression of MicroRNA in synovial fibroblasts and synovial tissue in rheumatoid arthritis. Arthritis Rheum.

[CR44] Szczepankiewicz D, Langwiński W, Kołodziejski P, Pruszyńska-Oszmałek E, Sassek M, Nowakowska J, Chmurzyńska A, Nowak KW, Szczepankiewicz A (2020). Allergic inflammation alters microRNA expression profile in adipose tissue in the rat. Genes (basel).

[CR45] Taheri F, Ebrahimi SO, Shareef S, Reiisi S (2020). Regulatory and immunomodulatory role of miR-34a in T cell immunity. Life Sci.

[CR46] Tili E, Michaille JJ, Wernicke D, Alder H, Costinean S, Volinia S, Croce CM (2011). Mutator activity induced by microRNA-155 (miR-155) links inflammation and cancer. Proc Natl Acad Sci USA.

[CR47] Vilmont V, Tourneur L, Chiocchia G (2012). Fas-associated death domain protein and adenosine partnership: fad in RA. Rheumatology (oxford).

[CR48] Wang C, Zhang C, Liu L, Xi A, Chen B, Li Y, Du J (2017). Macrophage-derived mir-155-containing exosomes suppress fibroblast proliferation and promote fibroblast inflammation during cardiac injury. Mol Ther.

[CR49] Wang XY, Yuan L, Li YL, Gan SJ, Ren L, Zhang F, Jiang J, Qi XW (2018). RNA activation technique and its applications in cancer research. Am J Cancer Res.

[CR50] Wang (2021). microRNA-26a directly targeting MMP14 and MMP16 inhibits the cancer cell proliferation, migration and invasion in cutaneous squamous cell carcinoma [Retraction]. Cancer Manag Res.

[CR51] Wang X, He Y, Mackowiak B, Gao B (2021). MicroRNAs as regulators, biomarkers and therapeutic targets in liver diseases. Gut.

[CR52] Wen DY, Huang JC, Wang JY, Pan WY, Zeng JH, Pang YY, Yang H (2018). Potential clinical value and putative biological function of miR-122-5p in hepatocellular carcinoma: a comprehensive study using microarray and RNA sequencing data. Oncol Lett.

[CR53] Yan L, Hu F, Yan X, Wei Y, Ma W, Wang Y, Lu S, Wang Z (2016). Inhibition of microRNA-155 ameliorates experimental autoimmune myocarditis by modulating Th17/Treg immune response. J Mol Med (berl).

[CR54] Yu G, Song Y, Xie C, Tao L, Wan F, Jiang L, Wang J, Tang J (2019). MiR-142a-3p and miR-155-5p reduce methamphetamine-induced inflammation: role of the target protein Peli1. Toxicol Appl Pharmacol.

[CR55] Yu H, Lin L, Zhang Z, Zhang H, Hu H (2020). Targeting NF-κB pathway for the therapy of diseases: mechanism and clinical study. Signal Transduct Target Ther.

[CR56] Zhang L, Zhu G, Mei L, Wu C, Qiu L, Cui C, Liu Y, Teng IT, Tan W (2015). Self-assembled DNA immunonanoflowers as multivalent CpG nanoagents. ACS Appl Mater Interfaces.

[CR57] Zhang Y, Wu X, Wu J, Li S, Han S, Lin Z, Ding S, Jia X, Gong W (2019). Decreased expression of microRNA-510 in intestinal tissue contributes to post-infectious irritable bowel syndrome via targeting PRDX1. Am J Transl Res.

[CR58] Zhang J, Lan T, Lu Y (2019). Molecular engineering of functional nucleic acid nanomaterials toward in vivo applications. Adv Healthc Mater.

[CR59] Zhang M, Wang L, Huang S, He X (2020). MicroRNA-223 targets NLRP3 to relieve inflammation and alleviate spinal cord injury. Life Sci.

[CR60] Zhu G, Hu R, Zhao Z, Chen Z, Zhang X, Tan W (2013). Noncanonical self-assembly of multifunctional DNA nanoflowers for biomedical applications. J Am Chem Soc.

