# Supplementary material for: Preparation of a miR-155-activating nucleic acid nanoflower to study the molecular mechanism of miR-155 in inflammation
Source: Mol Med. 2022 Jun 17;28:66. doi: 10.1186/s10020-022-00495-4 (PMC9204882; doi:10.1186/s10020-022-00495-4)
Supplement: Supplementary file 1 — Additional file 1. Host gene MiR155HG and the 10,000 base sequence upstream of the 5'end of the first exon. [file 10020_2022_495_MOESM1_ESM.docx]

**SUPPLEMENTARY DATE-1**

Host gene MiR155HG and the 10,000 base sequence upstream of the 5'end of the first exon

CAAGGGGATTATATTTCAATATGAGATTTGGGTAGCGACATATATCCAAACTATATAAAGATATGCCAAATATACCAAACACTGTATTTATTAACATTCATAGACCCTTAATTTGTTGAGTCAAAATTGCACCCCTGTTTAGGTCATCTTTGAATATATACACTTGCTATTCTTCTGTTGGCCTCCTGTTTCCTAGCAAAGTTAATTTAATTCAGAATTTATTGATTAACTACCTTGCACCTAACACTAAATACTATGCTAAGTATTATGAGGAATAGTAATTTAGTAAATAAAACATGAATCCAGTTCTTGAACATGTTACATTTATGTTAGCAAGATAAAAACAAACACTGATAATGCAGCTCAATAAGAAAATCAAATGATATAGTATGAAATGCAACAGTTAATAATAGAGAAATCTGGGCCAGATGCAGTGATTTATGCCTGTAATTCCAGCACTTTAGGTGGCTGAGGTAGGAGGCTCACTTGAGGCCACGAGTTCAAGACCAGCCTGGGCAGCATAATAAAACCCTGTCTCTACAAAGAATAAAAATTTAAAAAAAAATTGGTTGGGCATGGTGGCACATGCTTGTAGTCCTAGCTACATGTGAGGCTGAGATGGGAGGATCACTTAAGGCTAGGAATGTGATGTTTCAGCGAGCTATGATTGTGCCACTGCACTCATCCTGGGTGACAGAGAGAGACCCTGTCTCTAAAATAATAATAAAGAAATCTGACCATCAGTGCCATAAGAGTTTGGACAAAAAGTTTAGACTATGAAAGCTGGATAAGTCAGGGAAGATTTTTTTAGAAGAATAAATATATAGAACTCCAACAAAGAGCTGTGTTCAAAATACAGGATAGAAATCAGCATGACTAAAGTTTTGTTCTCTGATGATATCAAAGATATTGGTCACTTCTACAAAAAAAAAAAAAGAATTTTAACTACACATTCCTTTGAGGCTTGAAGAAAACGTGGGAAACTGAGATATTCCTTTTGTGTTTATCTACCTGAAAGCATTTTGTTATTTCAGAGATAACTGAGCCTGGAACTTATATAGATGCATACGTTTTCTGTAAAATAGAATAATGTCAAGTGACCTCTTGGGTATGTTTGTATTAGTTTCGTAATGCATTTGTTTACACGAATGATAATCTTGAAGCTCAAGGTTAGCTCATTTCCACAGTTTACTGCCCCTATTCATGCTACTCCCTTGGATTCACTTTGTCCCACACCCTAAACCTAGCACCGGGATAAGTTTAAAATTATATTCAACAGAATTAACATTAGAATGCTGTTCTTAAAACAGACCTGGAGATGCAAAAAAGGATTTAAAAGGCCATTGGGATAGCTGAATAATTTAAAAATAAAGCTTGGTAAAAAGAAAACTGAAATTATTGTATTTATTACAAAAAGCATACACACCTGTAGAAATCATAGATGAGGCCCCTACAGAGACTGGTGAGATGCTTCTGAGCAAGGACCCATGAGGGTTGCAATTGCCCAGAGGTATAGAGGATACCATTCTGAGGTCTCCAAAGATGGTCCTCACCCCTGTATGTTTCCCCTATAAATACTTTTAAAAACCTGAAAAAAATGAAAAAGAAGAATACAAAAAGAAAGCCAAAAACCACTTTCATAGTCAGAGAGTGTTGTGGACTGAATTGTACCTCCCCTCACCTAATTCATATGTTGAAGCCCTGACCCCCAATATGATTGTATTTGGAGACAGGGCTTTTAGAATTAAGGTTAAAATGAGGTCAGAAGGATGGTGTTCTAATCCAATAGGACTGGTGCCCTCATAGGAGGAGGAAGAGAGAGATCTCTCTCTCTAAATGCACACACTGAGGAAATTCCACATGAGCCCATAATGAGAAGGTGGCCATCTGCAAGTCAGGAAGAAAGCATTTCCTAGGCATCTAACCAGCTGGCACTTTGGTCTTAGACTTCCCATACTCCAGAACTATGAGAAGTAAATTTCTGTCTTTTAACCACTCAGTCTATCATATTTTGTTATGGAAACCCTAACGGGTTAATACAGGTTTTGATGCCAAGAAATGGAATGCTGCTATAACAAATACCTAAAAATGTTGAAGCAGCTTTGGAATTGAGTAGTGTGTAGGGGCTAGAAGAGTGTTAAGGTCCATGCTAGAAATACAGACATTAAAAGCAATTTCTGGTGAGGTTTCATACGGAAATAGAGAGTTGGAAAGAAAGCTTCGATCTTCTCAGTGAAAAGGTAAATACACATAAAGAGAACGTTGGCAGAAATATAGATGTTAACAGATATTCTGGTGAGGTCTCAAATGAAAATGGGGAACATTTTATTGGAAACTGAAGAAAAAGTTACCCTTGTTACATAGTGGCAAGGAACTTGGCTGAATTGTGCTCATGTTCTAGTGTTTTGTGGAAGACAGAACTTGAAAGCAATGAAACTGGATAGTTAACAGAGAATTTTTCTAAGCAGAGTGTTGAAGAAGTGGCTTTGTTCCTCCTGGCTGGTTTTAGGAATATGTGAAGGAGAGAGATTAATTGAAGAAGGGATTGTTGAGAAAAAAAGAACCAGACTTGAATATTAGGAAAATTCTCAGCCTACCCATACCGCAAAAAATGAGAAAGCATGCTCTGAAGAGAACATCAAAGTGTAGCTGGATTATCACCTCATAAAGAGCTAATGAGATTATATGAGTAAAAACATTTTCATTTTTAACTGAAGGGAACAGAGATGGAACAAAATGAAGGAAAGCTATTGAACTTCTTGGATTTGACAAGACAATAGAGCTATTTGGCTGCACACATACGCTATTCTTAAAGAAGAGGGGAAAATGACCCTGCAGGGAATCCAGAGGTCATCAGGGCTGCCACCTTGGTTTCTAAAGGTCAGATGGTCTCCACCTGAAGTCTTGGAGGCGAGACCTCCAACTAGAGCCATTGGGGTGATGCTGCCATCCCACTGGGTCTGAAGGACAGAGCATCAAACCAAAGAGGATTGTTCTTGAGCCTTTAGACCTAATGGAGTTTGACTCACTAGATTTTTGGACTTGCTTGGGACCTATCACCTCTTCCTTCTTTTCTACTTCTCTTTTTTGAAATGGAAATGTTTACCCTATTCCTGTCTAACCATTGAATTCTGGAAGCACATAACTTATCTGACTTGCCCAGGTTCACATCTGGAAAGGAATTTTGCCTCGTGATGAATCACAGCTGGAGTCTTATCCATATCTGATTTGTAGGACATTTAGATGAGACTTTGGACTTTATGTTGGACTTTTGTGGGCTATTGAGTTAAAATATATATTTTTTGCATGTGAGAAGGAAGTAAATTTGGGAGGCCATGGGCAATATGCTATGATCTAAATTGTGTTCCCCCACAACGTTGGTGTTCCTACCATCAACGGTGGATTGGATAAAGAAAATGTGATATATATACACCATGGAATATTATGCAGCTATAAAAAGAAAAAATCATATCTATTGCACAACATGAATGCAGCTGGAGGCCATTATCTTAAGCAATCACCAATAAAGCCCTAATCACCAACAGAAATTTGGAGATGGGGCTTTTAGGAGGTAATCAATGAGGTTATAAGGCTAGGGCCCTAATCTAAACAGGAGTAGTGGCATTATAAAGATAGGGAGAGAGGTGGGTATATCTCTCCACATGCACCCACCAAGGAAAGGTCATATGAGAACATAGCAAGAAGGTGGTTGTCTGCAGGAAGAAAGTGCTCACCTAGAACTGAATCAGCACCTTGATCTTAGGCTTCCTGCCTCCAGAACTGTGAGCAATAAATTTCTGTTATTTAAGTCATAGTATTTTGTGACAGTCCTAGGACTAATTCAATGAGCAATATGAGAGTATCACTGAGTTCTCAGCTTGGTTCCAGGTGCCTGACTTATACATGTTGGTCTTCAGGCAGAAAAAGAACACAAGAATAGACACTGCTGTCTCAAACTGTAAAAATTTTAGAAGTAAACCTAGATATCAAATTGGCAAAGAATTTGTGGCAAAGTCCTCAAAAGCAGTTGCAACAAAAGCAAAAATTGACAAATGGGACATAACTAAATTAAAGAGCTTCTGCACAGCAAAAGAAACTATCAACAGAGTAAACAGATGATAGAATGGGAGAAAATATCTGCAAACTATGCTTCTGACAAAGGTCTAATATCCATAATCTAAAAGGAACTTAAACAAGTCAATGAGCAAAAACAACCCCATTAAAAAGTGGGTAAAGGACATGAACAGACACTTCTCAAAAGAAGTCATACAAGCAGACAACAAACATATGAAAAAATGTTCATCACCACTAATCATCAGAGAATGCAACTCAAAGCCACAATGAGATACCATATCACACCAGTCAGAATGGCTATTACTATAAAGCCCCAAAATAATAGATGTTGGTGAGGCTGCAGAAAACAAGGGAATGACTATACACTGTTTATGGGGATGTAAATTAGTTCAGCCACTGTAGAAAGCAGTTTGGATATCTCTTAAAGAACTAAAAATAGAACTACCATTCAACCCAGCAATCCCATTCTGAGTATATACCCAAAGGGAAATAAATTGTTCTACCAAAAAGGCACATGCACTTGTATGTTCACTCCAGCACTATTCACAATAACAAAAACATGAAATCTACCTAGATGTCCATCAATGGTGGATTGGATAAAGAAAATATGATATATACACACCATGGAATACTATGCAGCTGTTTAAAAAACCAAAATCGTATCTTTTGCACAACATGGATGCAGCTGGAGGCCATTATCTTAAGCAAATTAATGCAGAAACAGAAAATCACCTACTACATGTTCTCACTTGTAAGTGGGAAATAAACATTAAGTACACAGGGCCGGGCATGGTGGCTTATGCCTGTAATCCTAGCACTTAGAGAGGCCGAGGCGGGTGGGTTACTTGAGGCCAGGAGTTCAAGACCAGCCTGGCCAACATGGAGAAACCCTGCCTCTACTTAAAAATACAAAAATTAGCCTGGCGTGGTGGTGCACACCTGTAGTCCCAGCCACTCGGGAGGCTGAGGCATGAGAATTGCTTCAGCCTGGGAGGCAGAGGTTGCAGTGAGCACTCCAGTCCAGGCGACAGAGCAAGACTTTGTCTCAAAAAAAAGCGGGGGGTACACATGGACATAAAAATGGGAATAATATACACTGGAGACTACAAGATAGGGGAGGAAGGGAGCGCAGCATGGGATGAAAAATTGCTTAGTAGGTATTATGCTCACTACCTGCGTGATGGGTTTAATTGTACCCCAAACCTCAGCATCACACAATATACCCTTATAACAAATCTGCACATGTACTCCCTGAATATAAAAGTTGAAAAAAATAATAGACACTGTTGGTGCTCAGAGAAGTCTCCCCAGTTATTTCAGAGAGGAAGGGGGTGGGAAAGACAGTGTAGAGTAATTTACTACAGGTGACTCATCAATTCACAAACTGAACCTTTAGACACTGAGCCAGGCCAGCTGAAATAAGAATATGGTTGAGGTCAGATACTGAGGGAAGTGAGTGCTATGGGGGGGCAGGTGGAGGAATTTTCTCTATATGTGATGTGATGATGATGACATCTATATGGAAGCAGAATGGTTCCCTTCCAAGGTTGTTCAAGAACCAAAATATTTTGAGAATCTTCGTCCTTTCCCCAGAACCTAACTTTTCCTCCATTATATGAAAATTTCCAAAAGACTGTGAAAACCTACCTTCCCAAAATGTTCAAATGAAGAAAATGAGCCAAGAGCAAAATCTCTACACTACCAAAAACTTTAAATTTTATGAAAAAAGTTATCTATGGAAGGCAGTGTATGTATAGACAAAAGAATTCTTAAGAGGCATTTGATGAGTATAAATGACTAAACAATTAGAGCATAGCTAAAAAGAGTCTGTAACTCACTGCAGAAAACACTAGTCTCCTTTGCAGAGGTTTGACTTTATTCCAGTGTTCCTTATGAAGGGGTGAATGGGAAAGATAAGGTTGAAAGGTTCATTCTCTCTAATTTGTAAATTTCATCTATTTGATAGAAAGAGAGAGAAAAAAAGCCACTCCTTTGCCCTCTGAAGGGAAAGGCATCCCAGCATTTAAGAGTATCACATAACACTCCAAAGCAGCAACCCATTTACAGAGGAAACATGAGTGAAAACAACATAGGCCAGAATTGGGCTTCCAAATGTTATAAACAGTGAAAGGAATAAAAATTAAATTATGATAGGAAAAAATTCTCTGATGCTTTGTTTGTGAAAAATAAAAACAAACACTCTTCATGAAATCTGAACATCTTATGAAGGCTACATTTTTTTAGCCTCAGGCATTAATTATGGTTGCGCCACTGATGGAGTCCAAAGGAAGCCAATTCAATCCACTTCATCCTCAGGGATGGAGAGCAACCACAAGTTTACAAAATAATTAATTATCTCCAGGTTATTGTACAACAGGGTAAACAGTGAACTGAAACCCTGTACCACAATAAATAAGCTGGTCAAGGCTGAAATGTGGGTCTATTTCTTCACTCACCATCTCTGTCTTACATTCTTCCAAAGGGCTGAACTTCAAGATCGTTGAGTACTAAACCTCCAGAAACTATTTTTATTTTTTTCTACCACTTTTTTTTTTTTAAGGAGAGAGATTGGAGGCTGGTTCCTAATCATTCCCTGTGATTATTGTAGGTTTCTGTAGAATTTCATGAAACTATGATGTTTTTAGCAAGACTTTGTCATTGATCTGTAGCTGTGCCAGTTGTCACAATCTATTCATCATTATTGATAATAAGTAATATCAAGAAGTACCACTCTTATCTGGAAGGTCATTGATGTGAGCTGCTCGGTCTCTGGAGGGTAAGATGAGGGATGAGAACACCAGCCCCATCAGCCCTGGAGACACATCCATATTACTTTGCGAGGGCTGCTGTGACAAGAGTACCGCAAACTGGGTGGTTTAAACAACAGAAATTTATTGTTTTTCAGTTCTGTGAGCTGAAGTCCAAGATCAAGATGTCAGCAGGACTGGTTCCTCCTGAGAGCTGTGAGGGAAGGATCTGTTTCAAGCCCCTCTCCTTGGCTGGTAGATGGCCATCTTTTCCCAATGTTTCTTCACACTGTCTTCCTTCTATATGTGTTTCTTTCTCTACACATCATGTTCTTTTTCTAAGAACACCAGTCATATTGGATTAGGAGCCAACCCTATTTTAATAAGACCTCATCTTGACTAATTACATCCACAAAGGCCTTGTTTCCACATAAGGTTATATTCTGAAGTACTGGTTGGTCTACAGCCATACCACCCCACACACACCCGATCTCATCTGAAGTACTGGGTGTTAGGACATCAATATATGAATTTGGGAGGTGGGGGGAGGGGGACACAATTCAGCCCACAACAACATCCATGGGGTACTTGTCCCGGCAGGTGGCCAGCTCTTACTTTCTCAAGCTATCCATTTGGAGCAGGTCCTTTTTTTTTTTTCTTTTTTTTTTTTTTTGAGACAGTCTCGCTCGCATTCCAGGCTGGAATGCAGTGGCGCGATCTTGGCTCACCGCAAACTCCGCCTCCCGCATTCATGCCATTCTCCTGCCTCAGCCTCCAGAGTAGCTGGGACTACAGGTGCCCGCCATCATGCCCGGCTAATTTTTTTTTCTTTTTTGTATTTTTAGTAGATTCAGGGTTTCACCATTCTAGCCAGGATGGTCTCAATCTCCTGACCTCATGATCCAGTCACCTCAGCCTCCCAAAGTGCTGGGATTACAGGATGAGCCACCGCGCCTGGCCTGGAGCAGGTACTTTTTATCCATCACCTTATTTAATCATCATATCCCTCTTAGTCTGCTAGGGTTGCCATAAGAAAATACCATAGACTGGATGGCTGATACAACAACAATTTATTTTCTCACATTCTGGAGGCTAGAAAGTTCCAGATCAAGGTCCTGCAGGGTTCGGTTTCTGGTGAGGACTCTCTTCCTGGCTTACAGGAAACCACCATCCCATTGTGTGCTCACATGACCTCTGCTTTGACTGATTTCTGAGAGAAAGGGAATTCTGGTGTCTCTTCCTCTTCTCATAAGAGCACTAGCTCTATGGGATTAGTGATCCACCCTTTTGACCTCATTTAACCTTTATCACCTCCTCACAGGCCCTATCTCCAAATGGGGATAACATTGGGGATCAGGGCTTCAACATATAAAGTTGAAGAGAACATGATTCAGTCTATAGTAATCCCTGATGGGAGACATCATTATTGTCATTTACAGGAAGCTGAGACATAAAAAGTTTAACGTTCAGATTCCACAGCTAGAATTAGTGTAAAATGCAGGTGTATTTGACTCCTAAAACCCATTTATCCATCCACTATTCTGGGTGTCTCAAACTGGGACCTAAAATGCGGAGGTTTGCACATGACCTGAAATTACTTCCACCACTGGGAGAAGGGAGGATTGAGCAATTTCCTGTTTCTATTAAAGTGCATTATACAAACTTGTCATTCTGGGGATGAAAGGTCACCCTAGAATTGCCTATGGGCAATTTCTTATAGTTCAACCTAGAATGAGAAATGGGAAATTCAGAAAGGCATTGTAGGCATCTGTAACCAGCAGAGGGACGTGCCCCACCTGGGTGGGGACCATGCATCCTTGCCACATGCCCCACTGCACAACTTCCCCAGCTCCTCAACGTCACATGGATCTGGAAAGCAGGGAGACTGGACTACGGAGCCAGCCCTCCAGGGTTGGAACTGAGTTTGAATCTCAGCTTGACTACTTACTAGGATCTAAGGTAACATGTTTAATCTCTCTGTGCTCAGTTTCCTCATCTGGAAATAGAGATTATAATGCTCCTCCCTCATATGATCATAGTGAGGACTGAAAGAGTTAATGCATATAAGGAGTGTTAGGACAGGATCTGGCACATGGTAAATGCTTTATAAGTGTTAGTTGTTATCATCATGATTATTCTTGTTTGAATTATAAGAGAAAAAGCATGTATCTTAAGAATAGAGGGTTTTAAAATGCTCCCAAGTTCCTTAACCAACCTGAGCCATCTGTAAATTAAGTACTATGGGATTTCCAGCTCTGACATGTATTCTGACATGTAACTGGCATCAGTTTAATACAAACTATTCTAAAATGTCTTGTGCCCTTGACAGGGAGGTCCAAGTACTGGATACTTGCAATGCAATCCAGCAGTGGCCCCGTCTTTCTTACAAAAAGGCCCCAGTCACATGTTGATGAGGCTAGATCTATTCCTGTCCTCCTTCTCTTCCCCATATTTCCTTATTCTTCTTCAAGTCTAAACGTTTATTGAACTGAGACCCATGAATGAGTTACTCGACTAGGCTTGTAGGATAAACTTGCCAGGCTTTAGATCTATTTGTTCCTTTCCATCCCCCAAAATCAAATGGCTGCTCCAGGAAAATGTTCCCCTTGTGGCAGGGTCCGGGAGAAAAGAGAGAAGCGACAAAACCAAAAATTAAAACGACCGAAGTCCCATATGCTCCAGGAATATGTCCTGGAGATGGGAGTGGAGGGCAGGGGGAGAATGTTGTTGAGGTCAAAATTTTTGAAGTTTTAAGTCCTATATCTTGACATCCCGAGTATAAATGCGGGTACCAGACACAGTACAAACGTTCTCAAAGCCCAGTTACGTATTCCAAACCAAACGCGGGCTCTTGAAGGGTGATGAGGTAGGGATGAAATCCAGGATCGCCTGAAGACCATTTCTTCCTCTCTTAGGGACCTGCTGGTCTCCAGCTGATTCGGTCCAGGAGGAAAAACCTCCCACTTGCTCCTCTCGGGCTCCCTGCAAGGAGAGAGTAGAGACACTCCTGCCACCCAGTTGCAAGAAGTCGCCACTTCCCCCTCCAGCCGACTGAAAGTTCGGGCGACGTCTGGGCCGTCATTTGAAGGCGTTTCCTTTTCTTTAAGAACAAAGGTTGGAGCCCAAGCCTTGCGGCGCGGTGCAGGAAAGTACACGGCGTGTGTTGAGAGAAAAAAAA
