# Supplementary material for: Preparation of a miR-155-activating nucleic acid nanoflower to study the molecular mechanism of miR-155 in inflammation
Source: Mol Med. 2022 Jun 17;28:66. doi: 10.1186/s10020-022-00495-4 (PMC9204882; doi:10.1186/s10020-022-00495-4)
Supplement: Supplementary file 2 — Additional file 2. Gene INPP5D and the 5000 base sequence upstream of the 5'end. [file 10020_2022_495_MOESM2_ESM.docx]

**SUPPLEMENTARY DATE-2**

Gene INPP5D and the 5000 base sequence upstream of the 5'end

AAGGGATCATAATCTGCTAGTTTGGGAGTGCTCCAGCTCTGCCAGACGTCTCCAGTGGGACGGTCTCCCTTCATCCTAGTCAGGGGCTGGGACTTGACTTCCTCTTCCACCATCTCAGTAAGCGACCTTGGCCTTTGATTTGAGAGGGGCCGATGTGGGCAGGGTAGATTCTCAGGACACAGGAGAGCATCCATCCTTCTTAACAAACTGTGAATAAGGTGCTGGCTCCTGCTTTTGACTGGTTTTCCCGGCTGGTCTTCCTCAGTGGCAGGAAGCAGGAGCCTGGGGGCTGACCCACCTGCCCTGGGCTCCTTGGGAGGGGAACAGTGGTGCTTGATCCCAGGAGGCTCCCTGTGAGTGGCTGCTCCCTTCTCCAGCAAAGGATGTCTTATTATCAGGTCTGAAGATGAGGGAAGTTCACTATTGATTACGCCAGCCAGTGAGCAACTTTCTAACTTCACGGTCAAGTATTTGCCCTTCTATTAATCAGGGTTCTTCAGAGAAGCAGATAGATAAGTAGATAGATTAGATAGATAGGGAGATAGATGGCTAGATAGATAGGGAGATAGATGGCTAGATAGATAGATAGATGGCTAAATAGATAGATGGATGGCTACATGGATGGATAGATAGATGGCTAGATAGATGGATGGATAGCTACATAGATGGATGGATAGATAGATAGATAGATAGATGGCTAAATAGATAGATGGCTAGATAGATAGACAGACAGATGGCTAGATACATAGACAGATGGCTAGATAGATATATGGATAAATAGATGACTAGATAGATAGATGACTAAATAGATAGGCAGATCGATAGATTTATAGATCAATAGATAGATAGGTAATAGTTAGACAGATGGCTAGACAGATGGATGGCCAGATAGATAGATAGATGGCTAGATAGATAGTGACTAGATAGATGGATAGGTAGATGGCTAGATAGATAGATGGATGGATGGTTAGCTAGATAGCTAGATAAATGGCTGGATAGATAGATGGATGGCTAGATACATAGATGACTAGATAGATAGTGGCTAGATGGATAGATAGATGGATGGCTAGATAGATGGCTAGATAGATAAATAGATGGATAGATAGATAGATGGCTAGATAGGTGGATAGATAGGTGGATGGCTAGAGAGATACACAGATGTATGGATAGATAGATAGATAGATAGATAGATAGATAGATAGATAGATAGATAGATATGGAATTGGCTCATAGGATGGTCAAGGCTGGCAAGTGCAAAGTCTGTAGAGTGGGCTGGCAGGCTGGAGACCCCGGGAAGAGTTGATGCTGCGGCTCGAGTCCATAGGCAATCTACTGGCAAATTTACTCTTTGGAGGAAGTCAGTCTTTTTCTATTAAGACCTTCAGCTTATTGGATGAGGCCTACACACAGGAATTGCTTTACTCAAAGTCTACTGATTTAAATGTTCATGTCATCTAAAAAATACTTTCAGAGAGACATCCAGAATAATGTTGACCAAATATCTGGGTCCTGAGGCCCAGCCAAGTTGACATAAAATTAACCATCACTGCTCCCTTTGCTTTTAGCACTCAGTGAGTGAACACTTTCAGCCCCTGCACCCCAAGCCATGAGTGATATCTATCTATCCACCCAGGCTGGGGGGAGTGGGAGCCCTGGGGCATAGTAAAGGGCTTCGATGAACACAGGAGGGGAAGGGAAGTACAGAGACAAATGCTTAGATATATTGTATATCTGTTAACATTGTATAGTAAATATATATTATATATATATAATGTGTGTGTATATATATACACACTATATATATGATGTAATGTGTGTGTGTATGTGTGTATATATATATATATATATACACACACACACACACATATATATATACAGTATATATTGACCTCTGCCTCCCCTCTCCTGGCTCAGTCCCTTCTCAGACCCTGAAATTCTTTCTGTTTCTTGTCCACGGTGGAAGGACCCCTGGACAAGAAGCTTGGTGTCTCCATTGTCCCTCTCTCCCAGCCTGCCCTGACCCTGTCCTGAGACTCAAACCTTCCTGTGACCCGCCCTCACTTAAGGTTGCCAAAGCAGAATTGCTCCAATGCCTTGGCCCTGCCATGTCAGCCCTCCTCTTCAACACTGAGGCCTGTCTGTTCTTCCCGGAGCTTCTAGAGTTGGCTGAGTGCCCATGACCAGGTGCCTGCCAGACCACCCTGATGCTTCTCACTGCCCAGCTTTCCCAGAAATTGTTCAAGTCCCCTGATGGGGCCTGGGGCTTGCCAGGGCTGGCAGTGCCTTTGGGCAGGTGCCCCAGTCTGGACCCAGGATCACATGAGTGAGTCTGACCCCACTTCTTCCCCTCAGCTGCTTTCCAAGCCTCTGCCCCATGGGTGAGGTTGACCAGATGCTCCAAGGAATGCATAGACTCACACTATGCAATCGCTCTGGGGCCCTATGGCTGGGTGTGACTCCTGGGCACAGCCTGGGGAGCAGGTTTCTCCTTTGAGTGGCCCCGAGCATTGAGGACAGCAAGGATAGTGGCAGGTGGTCTAGGCCCTCATCACCCCTGCCAGCAGCAGCTGGCAGTCCCTTATCCCTTCTGGAGGCTCCTGTCCACTATCAGGCAGCTGCCTCCTCCAACGTCTTTCGAGGACATTGCTCCACAGCACCGATACACCCACGGGCTCTTCCCATCAGCATCATCTCCCACCTCCCATCCATGTGGTGATGCTGAGCTCTCCACATTGATCCTGCCAAGCCAGATGCAGGCAGACATCCCTGGAGAGTGAGACTGCTCTTGTATGTGGAGGGCCAGGGTGCAATCATCATCAAATGGGATCTCAAGGTAGGAATGCAGTGATTATGGCCTAACACATTCAAGAGAATATGGCTGGAAGATGTGTGGTTCTACCTTTATGCTATAAAATTTGGAACAGTAATTTTTTTTTTTTTTTACTGTGTCGCTCTATCACCCAGGCTGGAGTGCAGTGGCACGATCTCGACTCACTGCAATCTTCGCCTCCTGGGTTCAAGTGATTATCCTGCTCAGCCTCCAAAGTAGCTGTAGCTGCGATTATAGGCACGTACCACACCCACCTGATTTTTTTTTTTTTTTTTTTTAGTAGAGACAGGGTTTCACCATGTTGGCCAGGCTGGTCTCAAACTCCTGACCTCAGGTGATCCACTCCCCTCAGCCTCCCAAAGTGCTGGGATTACAGGTGTTAGCCACTGTGCCTGGCCACTCTCTAGCCATTATTTGAAGTCTTCATTGATAAATCACTATCCCTCACATGAGTTTGTGAATTTTGTAATACGTTAAAATTATTTATATTGACTGGAGACCTACAAAAAAAAAAAATCCACCCACCAAAAGGGCAGCTCTGATCTAGATACTGGGATTTCCTGAATGTGGAGGGCAGAAGGGTGGGGGACAAGCCAGCTGACCATGCTGGATAGATCAGGCAATTGGGTGGAAAAGGAAGCAAAGTTGGGATGGGCCTTAAAAATGAGGGAAGAGTGCCCGTGTGGCGTGTGTGCCAGGGGATCTCACTCTGTGGAATGGCAAGGACCGTGGGAATAGAGGTGTGAGAGGTGGGGGGCTGGGGGTTTGGGGGAAGCTCTCTAAGTAGGGCAGTCTTGGCATGGTAGGGGTCTCTAGAGATGAGTGGGTGGAGGTGTAAAGGGACCAGTATTGCAAATGGATCCTCCTGGTGGCGTGGTCCAGGACTTCATGGATGTTGGTGGAGGCCAGCAGGGAAGTGAGAAGGGTGTGAAGGTACACAATGGGAGCATAGGAGATGACCCCATAAGGAGGGGGTGGACAGGTGCTGAGTGGAGGCGTGGGTGGGGAATGAGAGGCCTCCACACTGGGCTGCTGCAGAGGTGGCGTGCTGCGGAAGGGCCGGAGCATGGAGGCCAGTTCCCATCACCACTCTATCCCCACGCCTGGCTCCCAGCCTGGCCAGAGGTCCTTGACTGGGAGTGCTTTGTCTGCCCTACAGACTCGCCTGCCATGTGTGTTGCTCAGCGGGGAGGTGCTGGGGGCCCTGTTCTGGGACAACCATCAGTGTGACACATGTTGAGCCCCTGGGCACATGTGAGCTCTTTTACGAGCTACGTCCTTCAAGTTAGACAAGAAGAAACCAAGGCCGAGAGAGAGAAGAAACTTGACCAGGTTCCCACAGCTTGAATGTGGTGGCGCTGCCTGGCTGGTGCCCCTTCCTGGAGGGAGTCTGTTGTCATGTCCTGGAGGCCAAGGCCTGGATGTGGGATGGTGGGGGCACTCTTTGTGCAGCGGGTCAACACGTGCCCCTTCGGCCTTGGTGGGCCGGACCCAAGGACGGAAGGTGCCCAGGTCTGGGGGCTGAGGGATGGGTGTCCACCGCAGAGCCCTCAGAAATGACTGGTCCTGGATGGCGGTCCCTGGAGAGGCAGCTCCTCTTTGAGTGGTGGGCATATGTGGCCATGCCCAGTCTTGGAGGCGGTGCAGTCCGACCGGCCACCCCGACTGCTGAGACGCAGGACTCAGCACCCAGTGTTGTCCTTGGCCAGGCGGCCAGCCTGCCCTGTGGGGCTTTGGGGTTCTCCTCTCCGAGCTTGTTTCCCCAGCATCCTGGAGAAGCCCAGGGAAAAACCCAGCCTATGTTCCCGCTGTAAACGGCCCACATCCCCTCCAAGCTCCCTTTGTGTGTCTCCAAGCTGGACTGCAGGGATCAGAGCTGTGCTGCCCGTGCAGCACCCACCCGCCCCTGGGAATGAGGGTAATGGGGTCACAGAGCCATCACTTGGACCTTGGCGCCCTCGGCTGGTGGCAGCCAAGGTCTCCCAGCACCCAGGCCGCTGGGAGTCTCTAAAGGGGAGGGGTGGGGCTCGACTGTCCCCTCCCCCTCAAGTTTGCTCTGTCCTGGGCAGGCCGTAGTCCCAGTTGAGAAGCTGTGCCCCCTTGGGTGTTTTGGAGGTTCAGGGTGGGTGCTGAAAAGCCCTGAGGGAGAGCAGAAGGCTCGGGGGCCTG
