# Supplementary material for: Preparation of a miR-155-activating nucleic acid nanoflower to study the molecular mechanism of miR-155 in inflammation
Source: Mol Med. 2022 Jun 17;28:66. doi: 10.1186/s10020-022-00495-4 (PMC9204882; doi:10.1186/s10020-022-00495-4)
Supplement: Supplementary file 3 — Additional file 3: Fig. S1. The secondary structure of the product after 1 time (A) and 5 times (B) of rolling circle transcription. Fig. S2. (A) The expression of miR-155 after 48h of LPS treatment of cells with different concentrations. (B)Relative expression of inflammation-related factor genes 72h after NFs (M5) treatment of cells (C) The speciation diagram of HUVECs cell line under normal culture. (D-I) The speciation diagram of the HUVEC cell line stimulated by LPS at 0.1, 1, and 10μg/mL for 12 h. (F-H) The speciation diagram of the HUVEC cell line stimulated by LPS at 0.1, 1, and 10μg/mL for 24 h. (*P<0.05vs Control; **P<0.01 vs Control). [file 10020_2022_495_MOESM3_ESM.docx]

Supplementary Figure 1：The secondary structure of the product after 1 time (A) and 5 times (B) of rolling circle transcription.


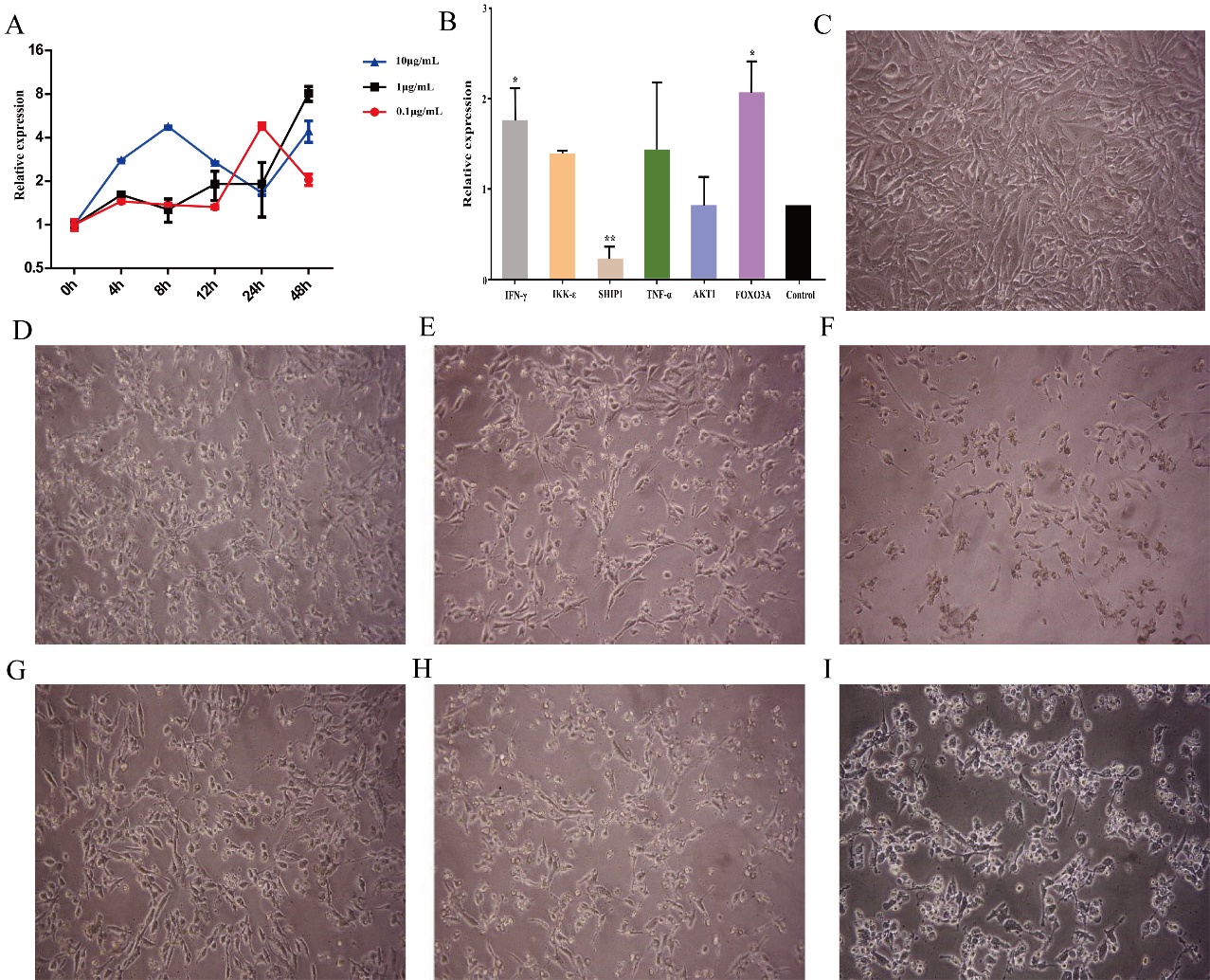


Supplementary Figure 2：(A) The expression of miR-155 after 48h of LPS treatment of cells with different concentrations. (B)Relative expression of inflammation-related factor genes 72h after NFs (M5) treatment of cells (C) The speciation diagram of HUVECs cell line under normal culture. (D-I) The speciation diagram of the HUVEC cell line stimulated by LPS at 0.1, 1, and 10μg/mL for 12 h. (F-H) The speciation diagram of the HUVEC cell line stimulated by LPS at 0.1, 1, and 10μg/mL for 24 h. (*P<0.05vs Control ；**P<0.01 vs Control）
