# Supplementary material for: Preparation of a miR-155-activating nucleic acid nanoflower to study the molecular mechanism of miR-155 in inflammation
Source: Mol Med. 2022 Jun 17;28:66. doi: 10.1186/s10020-022-00495-4 (PMC9204882; doi:10.1186/s10020-022-00495-4)
Supplement: Supplementary file 4 — Additional file 4: Table S1. Template and primer sequence information for preparing NFs. Table S2. RT-qPCR amplification primer information. Table S3 miR-155 target gene prediction results. Table S4. Enrichment analysis of target genes of miR-155. Table S5. Sequences in line with saRNA design principles. Table S6. Template and primer sequence for preparing nucleic acid nanoflowers that can activate SHIP1. [file 10020_2022_495_MOESM4_ESM.docx]

**SUPPLEMENTARY TABLE**

Supplementary table 1 Template and primer sequence information for preparing NFs

| Names | Sequence（5’→ 3’） |
| --- | --- |
| L-T | ATAGTGAGTCGTATTAACGTACCAACAAGCAACTCCAGTCCCAAATAACTTGTATTTGGGACTGGAGTTGCATCCCT |
| T7 | TAATACGACTCACTATAGGGAT |
| VEGF-706 S | GCAACUCCAGUCCCAAAUAdTdT |
| VEGF-706 AS | UAUUUGGGACUGGAGUUGCdTdT |

Note: The underlined part is the DNA sequence of VEGF-706 saRNA

Supplementary table 2 RT-qPCR amplification primer information

| Gene name |  | Sequence（5'-3'） |
| --- | --- | --- |
| GAPDH | Forward | GGAGCGAGATCCCTCCAAAAT |
|  | Reverse | GGCTGTTGTCATACTTCTCATGG |
| VEGF | Forward | ATGACGAGGGCCTGGAGTGTG |
|  | Reverse | CCTATGTGCTGGCCTTGGTGAG |
| SHIP1 | Forward | GCTGGAGGAAGAGGACACA |
|  | Reverse | AGTCAGCGGGATGTTTCTTG |
| IFN-γ | Forward | ACTGACTTGAATGTCCAACGCA |
|  | Reverse | ATCTGACTCCTTTTTCGCTTCC |
| IL-1β | Forward | AGGTGCATCGTGCACATAAG |
|  | Reverse | AAGCTGATGGCCCTAAACAG |
| IL-6 | Forward | AGGAGACTTGCCTGGTGAAA |
|  | Reverse | CAGGGGTGGTTATTGCATCT |
| TNF-α | Forward | ACTTTGGAGTCATTGCTC |
|  | Reverse | GGAAAGCCCATTTGAGTC |
| IKKɛ | Forward | TGCGTGCAGAAGTATCAAGC |
|  | Reverse | TACAGGCAGCCACAGAACAG |
| PI3K | Forward | TCCAGCACATGAACGTGTAAACAG |
|  | Reverse | CACACACTACATCAGTGGCTCAAAG |
| AKT | Forward | TGGGCAAGGGCACTTTCGG |
|  | Reverse | AGGCGGTCGTGGGTCTGGAA |
| FOXO3A | Forward | GCTGGAGGAAGAGGACACA |
|  | Reverse | AGTCAGCGGGATGTTTCTTG |
| NF-κB | Forward | CAGTGAGCGGAAGCGAGGAATG |
|  | Reverse | TGCCTCTCAGCCTGGAAGTCC |
| MiR155HG | Forward | TCAAGAACAACCTACCAGAGACCTT |
|  | Reverse | TCCTGGTTTTTTCCACCAT |
| miR-155 | Forward | AGTGCAGGGTCCGAGGTATT |
|  | Reverse | GCGCGTTAATGCTAATTGTGAT |
| U6 | Forward | CTCGCTTCGGCAGCACA |
|  | Reverse | AACGCTTCACGAATTTGCGT |
| miR-155 RT |  | GTCGTATCCAGTGCAGGGTCCGAGGTATTCGCA |
|  |  | CTGGATACGACACCCCT |

Supplementary table 3 miR-155 target gene prediction results

| Tool | The number of targeted genes | Top five target gene names |
| --- | --- | --- |
| TargetScan | 212 | EEF2、LRRC59、IRF2BP2、PAM、UQCR11 |
| miRanda | 265 | ASF1A、ARID2、HIVEP2、KAT2B、TYRP1 |
| PITA | 163 | BACH1、KBTBD2、POLE3、SDCBP、TRIP13 |
| picTar | 166 | ASTN2、BACH1、FBXO11、KBTBD2、MGC13272 |

Supplementary table 4 Enrichment analysis of target genes of miR-155

| signaling pathways | P | The Number of target gene enrichment | The name of the enriched target |
| --- | --- | --- | --- |
| PI3K-AKT | 1×10^-3^ | 9 | CCND1、CDKN1A、CSF1R、FOXO3、IL-6R、ITGB1、MYB、RELA、SHIP1 |
| TNF | 1×10^-5^ | 7 | CEBPB、ICAM1、MAP3K14、RELA、TAB2、TNF、VCAM1 |
| NF-кB | 5×10^-5^ | 6 | ICAM1、MAP3K14、RELA、TAB2、TNF、 VCAM1 |
| MAPK | 6×10^-3^ | 6 | MAP3K14、PAK2、RAPGEF2、RELA、TAB2、TNF |
| Ras | 0.022 | 5 | CSF1R、GAB2、KSR1、PAK2、RELA |
| T cell | 0.012 | 4 | MAP3K14、PAK2、RELA、TNF |
| FoxO | 0.026 | 4 | CCND1、CDKN1A、FOXO3、SMAD2 |
| TLR | 0.029 | 4 | RELA、SOCS1、TNF、TAB2 |

Supplementary table 5 Sequences in line with saRNA design principles

| Gene name | Positive-sense strand | antisense strand |
| --- | --- | --- |
| SHIP1-1 | GGA UGG UCA AGG CUG GCA A | UUG CCA GCC UUG ACC AUC C |
| SHIP1-2 | CAA GGC UGG CAA GUG CAA A | UUU GCA CUU GCC AGC CUU G |
| SHIP1-3 | GGC AAG UGC AAA GUC UGU A | UAC AGA CUU UGC ACU UGC C |
| SHIP1-4 | AGU UGA UGC UGC GGC UCG A | UCG AGC CGC AGC AUC AAC U |
| SHIP1-5 | GCU UAU UGG AUG AGG CCU A | UAG GCC UCA UCC AAU AAG C |
| SHIP1-6 | AUU GAA UGA GGC CUA CAC A | UGU GUA GGC CUC AUU CAA U |
| SHIP1-7 | GCA UAG UAA AGG GCU UCG A | UCG AAG CCC UUU ACUAUG C |
| SHIP1-8 | AAG GGC UUC GAU GAA CAC A | UGU GUU CAU CGA AGC CCU U |

Supplementary table 6 Template and primer sequence for preparing nucleic acid nanoflowers that can activate SHIP1

| name | Sequence（5’→ 3’） |
| --- | --- |
| Template-S1 | ATAGTGAGTCGTATTAACGTACCAACAAGGATGGTCAAGGCTGGCAAAACTTGTTGCCAGCCTTGACCATCCATCCCT |
| Template-S2 | ATAGTGAGTCGTATTAACGTACCAACAACAAGGCTGGCAAGTGCAAAAACTTGTTTGCACTTGCCAGCCTTGATCCCT |
| Template-S3 | ATAGTGAGTCGTATTAACGTACCAACAAGGCAAGTGCAAAGTCTGTAAACTTGTACAGACTTTGCACTTGCCATCCCT |
| Template-S4 | ATAGTGAGTCGTATTAACGTACCAACAAAGTTGATGCTGCGGCTCGAAACTTGTCGAGCCGCAGCATCAACTATCCCT |
| Template-S5 | ATAGTGAGTCGTATTAACGTACCAACAAGCTTATTGGATGAGGCCTAAACTTGTAGGCCTCATCCAATAAGCATCCCT |
| Template-S6 | ATAGTGAGTCGTATTAACGTACCAACAAATTGGATGAGGCCTACACAAACTTGTGTGTAGGCCTCATCCAATATCCCT |
| Template-S7 | ATAGTGAGTCGTATTAACGTACCAACAAGCATAGTAAAGGGCTTCGAAACTTGTCGAAGCCCTTTACTATGCATCCCT |
| Template-S8 | ATAGTGAGTCGTATTAACGTACCAACAAAAGGGCTTCGATGAACACAAACTTGTGTGTTCATCGAAGCCCTTATCCCT |
| T7 | TAATACGACTCACTATAGGGAT |

Note: All the above sequences (except T7) need to modify the phosphate group at the 5'end, and the underlined part is the DNA sequence of the saRNA selected.
